# Global impact of dietary risks on cancers: burdens across regions from 1990 to 2021 and the projection to 2035

**DOI:** 10.3389/fnut.2025.1585305

**Published:** 2025-07-04

**Authors:** Xiangrong Liu, Xinsheng Zhang, Yulu Wei, Zhimeng Yu, Lu Liu, Xin Qin, Yinghua Liu

**Affiliations:** ^1^Department of Nutrition, The First Medical Center of the Chinese People′s Liberation Army General Hospital, Beijing, China; ^2^People’s Liberation Army Postgraduate Medical School, Beijing, China; ^3^Yangzhou Maternal and Child Health Care Hospital, Yangzhou, China

**Keywords:** dietary risk, cancer, bibliometric analysis, global burden of disease, sociodemographic index, health inequity

## Abstract

**Background:**

The burden of cancer has been progressively increasing, with diet playing a key role in its development and progression. Previous studies on diet-related cancer burden mainly focused on individual factors or single cancer types. Therefore, this study aimed to analyze global trends in deaths and disability-adjusted life years (DALYs) associated with diet-related cancers from 1990 to 2021, predicting the burden up to 2035.

**Methods:**

Using bibliometric analysis and Global Burden of Disease (GBD) 2021 data, it examined mortality and DALY rates stratified by age, sex, cancer type, and region. Decomposition analysis assessed contributions of population growth, aging, and epidemiological transitions, while health inequalities were evaluated using inequality indices. Frontier analysis quantified gaps between current and minimum achievable burdens. Future trends for multiple tumors were projected using Bayesian Age-Period-Cohort (BAPC) modeling.

**Results:**

The age-standardized death rate (ASDR) for diet-related cancers declined from 12.24 (95% UI: 3.32 to 22.78) per 100,000 in 1990 to 7.90 (95% UI: 2.45 to 13.85) in 2021, and the age-standardized DALYs rate decreased from 302.48 (95% UI: 80.53 to 565.63) to 189.62 (95% UI: 57.13 to 335.37) per 100,000. In 2021, the highest ASDR were recorded in Central Europe, and the largest age-standardized DALYs rate were documented in Southern Sub-Saharan Africa. The greatest disease burden was identified in high-middle sociodemographic index (SDI) regions. Colon and rectum cancers were most prominent, with high red meat intake as the leading dietary risk. We also found that a higher disease burden was observed in males compared to females, and the burden increased progressively with age. Decomposition analysis revealed population growth as the main driver of increasing burdens, partly offset by epidemiological changes. Health inequality driven by economic factors has decreased. BAPC modeling predicted a continued decline in the future burden of multiple cancers, except for breast cancer.

**Conclusion:**

While the burden of diet-related cancers is declining, challenges remain, particularly in older populations and higher SDI regions. Persistent health inequalities affect esophageal and stomach cancers. These findings can guide targeted strategies for prevention and control.

## 1 Introduction

Cancer remains one of the most significant global health challenges, with its incidence and mortality rates on a continuous upward trajectory (1). The development of cancer is a multifaceted process, influenced by the interplay of genetic, environmental, and lifestyle factors. Among these, diet stands out as a modifiable risk factor, playing a crucial role in cancer prevention and control.

Extensive research has demonstrated that poor dietary habits can significantly elevate cancer risk and burden. For instance, diets high in red meat, processed foods, and added sugars have been linked to an increased risk of breast cancer, primarily through the activation of inflammatory pathways such as NF-κB (2). Conversely, adherence to a Mediterranean diet, abundant in vegetables and whole grains, has been associated with a 27% reduction in the risk of head and neck cancer (3). Additionally, a diet low in fiber can disrupt gut microbiota balance, diminishing the production of short-chain fatty acids and thereby fostering the progression of colorectal cancer (4). On the positive side, calcium-rich dairy products have been shown to offer protective benefits against colorectal cancer (5). In 2019, global data highlighted that 65,919 deaths from esophageal cancer were attributable to insufficient consumption of vegetables and fruits, representing 0.12% of all deaths worldwide. The age-standardized death rate (ASDR) for this cause was 0.81 per 100,000 individuals, with an age-standardized disability-adjusted life years (DALYs) rate of 19.24 (6).

Previous studies (7, 8) have predominantly concentrated on the effects of dietary risks on either individual cancer burdens or specific cancers within particular populations. This study utilized a multidisciplinary approach by integrating bibliometric analyses and the Global Burden of Disease (GBD) to conduct comprehensive research. According to the GBD data, five types of cancers were identified as being closely associated with dietary behaviors: esophageal cancer, stomach cancer, colon and rectum cancer, tracheal, bronchus, and lung cancer, and breast cancer. Initially, bibliometric analyses were utilized to identify key research trends regarding dietary risks and cancers. Secondly, this study provides a comprehensive statistical description and analysis of the mortality and DALYs burdens for these five cancers linked to nine dietary risks. Furthermore, it projects the trends in cancer burden up to the year 2035. This study represents the first epidemiological research to utilize the 2021 GBD data to examine the disease burden of multiple cancers attributable to dietary risks. The primary objective of this research is to furnish a scientific foundation for policymakers, public health experts, and clinicians. It underscores the importance of educating the public to improve dietary habits and refining cancer prevention strategies as pivotal measures to alleviate the global cancer burden.

## 2 Materials and methods

### 2.1 Data sources

The studies analyzed were sourced from Web of Science spanning from 1990 to 2025. The search query included (“Diet” OR “Dietary risks”) AND (“tumor” OR “Cancer” OR “neoplasm” OR “Carcinoma” OR “Sarcoma” OR “esophageal cancer” OR “stomach cancer” OR “colon cancer” OR “lung cancer” OR “breast cancer”) and inputted into Cite-Space for statistical assessment. Following the initial search, a meticulous screening process was conducted, retaining peer-reviewed articles on diet-related cancers and excluding irrelevant ones.

All data in this study were extracted from the GBD 2021 dataset, accessible via the GBD Results Tool.^1^ The GBD database represents a comprehensive global health resource, systematically collating epidemiological metrics—including incidence, prevalence, mortality, years of life lost, years lived with disability, and DALYs—for 371 diseases and injuries, alongside 88 major risk factors, across 204 countries and territories. From the 15 dietary risk factors evaluated in the 2021 GBD framework, nine were identified as being significantly associated with cancer outcomes. These comprised diets low in calcium, whole grains, milk, vegetables, fruits, and fiber, as well as diets high in sodium and red and processed meats.

The Sociodemographic Index (SDI) served as a composite indicator to evaluate socioeconomic development levels across nations and regions. This index integrates normalized metrics for per capita income, average educational attainment, total fertility rate, and population age distribution (9, 10). Based on 2021 GBD criteria, 204 countries and territories were stratified into five SDI tiers: low-SDI [0–0.47], low-middle SDI [0.47–0.62], middle SDI [0.62–0.71], high-middle SDI [0.71–0.81], and high SDI [0.81–1.00] (11).

### 2.2 Statistical analysis

#### 2.2.1 CiteSpace bibliometric analysis

CiteSpace, a Java-based visualization tool, detects research hotspots and predicts future trends by analyzing authors, countries, keywords, and other relevant metrics in the field. The knowledge graph was quantified through adjustments in node types, connections, and selection criteria for visual analysis.

#### 2.2.2 Global burden of diet-related cancers

This study employed two primary indicators to assess the burden of diet-related cancers: mortality and DALYs, both reported with 95% uncertainty intervals (UI). Temporal trends from 1990 to 2021 were analyzed using the estimated annual percentage change (EAPC) in age-standardized rates (ASR), enabling systematic comparisons across age groups, sexes, cancer subtypes, and geographic regions. Proportional distributions of diet-related cancers were evaluated at four key time points (1990, 2000, 2010, and 2021). Based on 2021 data, the contributions of specific dietary risk factors to the global cancer burden—particularly colorectal cancer—were examined across five SDI regions.

The spatiotemporal distribution patterns of diet-related cancer burden were characterized by ASR with corresponding 95% UI stratified according to the SDI (12, 13). The strength and direction of the association between SDI levels and diet-related cancer burden were evaluated using Spearman’s rank correlation model (14). The comparative risk assessment framework was applied to quantify the effects of demographic expansion, age structure transitions, and changes in disease incidence rates on temporal trends in disease burden. This factor decomposition methodology enabled stratification of total burden into constituent drivers, elucidating predominant determinants and their directional influences (15).

We used an age-standardized DALYs rate-based frontier analysis to assess differences in the burden of disease between different countries and regions that differed in SDI level. This approach focused on determining the lowest theoretically achievable ASR value for each country or region based on its current SDI level, thereby quantifying the difference between the current burden of disease and its theoretically lowest possible burden. For countries that had not reached the frontier, the distance from the frontier was calculated to reveal potential room for improvement. We used a combination of locally estimated scatterplot smoothing and local polynomial regression techniques to construct a smoothed borderline. To ensure the reliability of the analysis results, we performed 100 instances of self-help sampling (11).

Health disparities in age-standardized DALYs rate were quantified using two complementary measures: the slope index of inequality (SII), which assesses absolute inequality, and the concentration index of inequality (CII), which evaluates relative inequality across socioeconomic gradients (16). SII serves as a metric for quantifying absolute disparities in health outcomes across socioeconomic gradients. The CII is employed to assess the relative inequality in the distribution of health outcomes in relation to socioeconomic status.

We conduct Age-Period-Cohort (APC) analysis using the R package apc to assess the impacts of various age groups, time periods, and birth cohorts on disease burden (17). The Bayesian Age-Period-Cohort (BAPC) model effectively distinguishes the effects of age, period, and cohort on health outcomes, offering insights into temporal health trends across generations and the influence of public health events or policy shifts. Utilizing integrated nested Laplace approximations (INLA), a computationally efficient Bayesian method, it bypasses the lengthy simulations of traditional Markov chain Monte Carlo approaches. INLA rapidly delivers precise parameter estimates and predictions for large datasets by approximating posterior distributions. The model provides detailed prediction rates for various age groups and computes age-standardized rates to facilitate cross-population health comparisons (18, 19).

The data analysis and visualization were conducted using R software (version 4.4.1), with a significance level of *p* < 0.05 indicating statistical significance. Ethical approval was not necessary since the GBD database is publicly accessible.

## 3 Results

### 3.1 Bibliometric analysis

**Figure 1:**
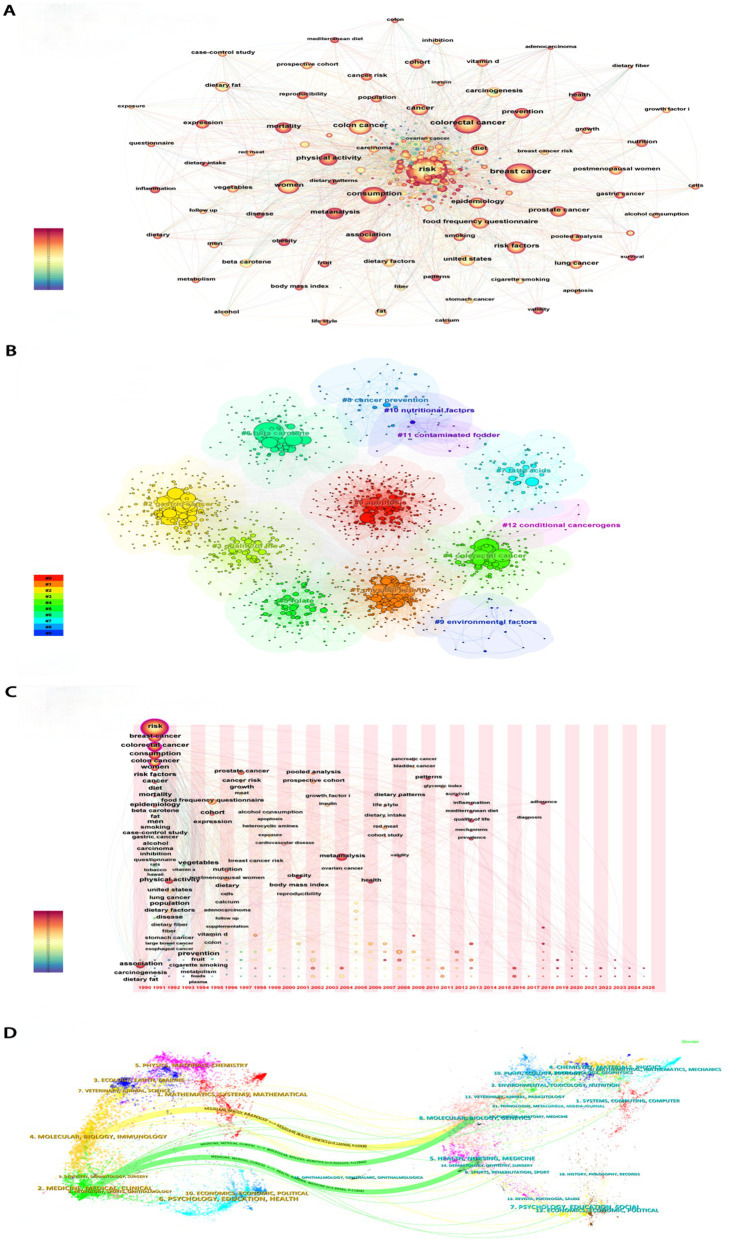
Bibliometric analyses results. **(A)** Co-occurrence network of Keyword. **(B)** Keyword clustering analyses. **(C)** Time-zone diagram. **(D)** Dual map of journals.

This study utilized a multidimensional visualization method to analyze research trends related to dietary risk factors and cancers. From 1990 to 2013, the number of studies exhibited an upward fluctuation, peaking in 2013, after which the level of research activity stabilized (Supplementary Figure S1A). The significant increase and deepening of research efforts highlighted the growing importance of these topics within public health policy. The United States played a leading role in this field, collaborating closely with countries such as China, Italy, France, and England to advance the development of this area (Supplementary Figure S1B). Keyword clustering analysis revealed that apoptosis, gastric cancer, colorectal cancer, and quality of life were key focal points in this domain (Figures 1A,B). Over time, research interests had broadened from diet-related digestive tract tumors to encompass other types of cancer, including lung, prostate, and pancreatic cancers. Additionally, fiber, vitamins, vegetables, and red meat had increasingly garnered attention. There had also been a notable rise in the emphasis placed on improving the quality of life for cancer patients. Furthermore, research in this field demonstrated a clear trend toward greater interdisciplinary and international collaboration, reflecting the global nature of addressing complex health challenges (Figures 1C,D). In this context, this article will further explore the tumor burden associated with dietary factors.

### 3.2 Burden of cancers attributable to dietary risk factors at the global level

Globally, deaths from cancers attributable to dietary risk factors increased from 463,979.47 (95% UI: 125,175.75 to 864,966.82) in 1990 to 669,656.23 (95% UI: 207,110.92 to 1,176,736.66) in 2021. Similarly, DALYs rose from 12,377,666.46 (95% UI: 3,287,466.84 to 23,260,291.81) to 16,403,608.55 (95% UI: 4,939,614.02 to 29,035,003.97) over the same period. In 2021, males experienced higher mortality (336,768.16; 95% UI: 116,797.83 to 612,379.37) and DALYs (8,338,902.95; 95% UI: 2,872,142.18 to 15,165,920.72) compared to females (deaths: 332,888.07; 95% UI: 94,639.01 to 578,474.26; DALYs: 8,064,705.6; 95% UI: 2,132,070.36 to 14,335,340.94).

**Figure 2:**
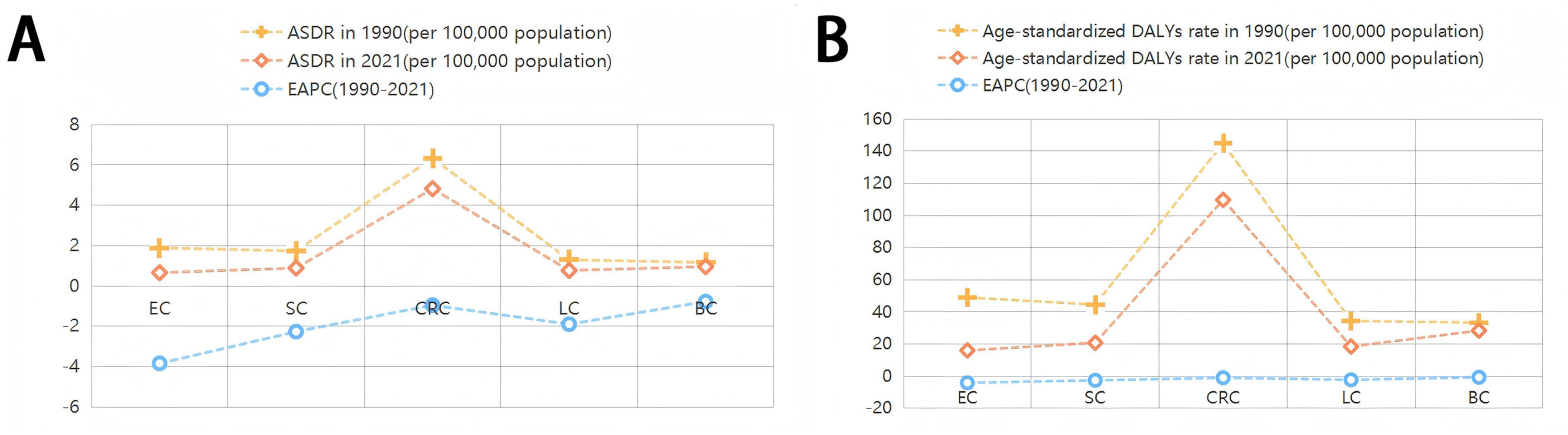
ASDR and age-standardised DALYs rates of cancers attributable to dietary risk factors in 2021 and their temporal trend from 1990 to 2021. EAPC, estimated annual percentage change; ASDR, age-standardized death rate; DALYs, disability-adjusted life years; EC,Esophageal cancer;SC, Stomach cancer; CRC, Colon and rectum cancer; LC, Tracheal, bronchus, and lung cancer; BC, Breast cancer.

The ASDR declined from 12.24 (95% UI: 3.32 to 22.78) per 100,000 population in 1990 to 7.90 (95% UI: 2.45 to 13.85) in 2021, with an EAPC of −1.54 (95% CI: −1.60 to −1.49). Correspondingly, the age-standardized DALYs rate decreased from 302.48 (95% UI: 80.53 to 565.63) to 189.62 (95% UI: 57.13 to 335.37) per 100,000 population (EAPC: −1.65; 95% CI: −1.72 to −1.59). Gender disparities persisted in 2021: males exhibited higher ASDR (8.73; 95% UI: 3.05 to 15.83) and DALYs rate (203.61; 95% UI: 70.44 to 369.27) than females (ASDR: 7.21; 95% UI: 2.04 to 12.52; DALYs rate: 177.90; 95% UI: 46.76 to 316.47). Notably, colon and rectum cancer showed the highest diet-related burden among all cancer types, with an ASDR of 4.82 (95% UI: 1.64 to 7.46) and DALYs rate of 109.71 (95% UI: 37.68 to 168.52) per 100,000 population (Table 1; Figure 2).

**Table 1:** ASDR and age-standardised DALYs rates of cancers attributable to dietary risk factors in 2021 and their temporal trend from 1990 to 2021 at global and regional levels.

	Death (95%UI)			DALYs (95%UI)		
	ASR in 1990 (per 100,000 population)	ASR in 2021 (per 100,000 population)	EAPC (1990–2021)	ASR in 1990 (per 100,000 population)	ASR in 2021 (per 100,000 population)	EAPC (1990–2021)
Location						
Global	12.24 (3.32, 22.78)	7.90 (2.45, 13.85)	−1.54 (−1.60, −1.49)	302.48 (80.53, 565.63)	189.62 (57.13, 335.37)	−1.65 (−1.72, −1.59)
Sex						
Male	13.86 (3.94, 27.19)	8.73 (3.05, 15.83)	−1.59 (−1.64, −1.55)	340.67 (92.81, 677.80)	203.61 (70.44, 369.27)	−1.78 (−1.84, −1.73)
Female	10.85 (2.79, 19.98)	7.21 (2.04, 12.52)	−1.48 (−1.54, −1.41)	268.62 (66.39, 502.20)	177.90 (46.76, 316.47)	−1.50 (−1.57, −1.42)
Aetiology						
Esophageal cancer	1.89 (−0.42, 3.73)	0.66 (−0.14, 1.38)	−3.83 (−4.04, −3.62)	49.01 (−10.74, 96.93)	16.02 (−3.37, 33.09)	−4.09 (−4.34, −3.84)
Stomach cancer	1.74 (0, 8.74)	0.89 (0, 4.37)	−2.26 (−2.35, −2.18)	44.53 (0, 222.31)	20.78 (0, 102.38)	−2.56 (−2.65, −2.47)
Colon and rectum cancer	6.33 (2.26, 9.47)	4.82 (1.64, 7.46)	−0.95 (−0.99, −0.92)	144.88 (53.10, 215.52)	109.71 (37.68, 168.52)	−0.98 (−1.02, −0.95)
Tracheal, bronchus, and lung cancer	1.30 (0.65, 1.91)	0.77 (0.40, 1.13)	−1.89 (−1.96, −1.81)	34.39 (17.24, 50.70)	18.46 (9.49, 26.90)	−2.23 (−2.32, −2.14)
Breast cancer	1.17 (0, 2.50)	0.96 (0, 2.06)	−0.77 (−0.82, −0.72)	33.31 (−0.01, 71.68)	28.37 (−0.01, 60.54)	−0.65 (−0.70, −0.60)
SDI Regions						
High SDI	13.81 (3.55, 24.16)	8.89 (2.23, 15.14)	−1.48 (−1.51, −1.45)	328.52 (81.48, 586.04)	207.54 (47.33, 352.53)	−1.53 (−1.55, −1.50)
High-middle SDI	14.79 (3.70, 28.86)	9.16 (2.87, 16.89)	−1.70 (−1.77, −1.63)	378.29 (91.34, 748.07)	219.57 (67.29, 406.47)	−1.95 (−2.04, −1.87)
Middle SDI	11.93 (3.11, 23.40)	7.22 (2.58, 13.46)	−1.83 (−1.93, −1.73)	309.73 (77.77, 611.05)	180.58 (60.55, 336.14)	−1.95 (−2.08, −1.83)
Low-middle SDI	5.30 (2.06, 9.75)	5.23 (1.94, 9.00)	−0.04 (−0.07, −0.01)	143.02 (51.42, 265.59)	139.67 (46.84, 242.91)	−0.08 (−0.11, −0.05)
Low SDI	6.61 (2.53, 11.54)	5.81 (2.25, 9.60)	−0.53 (−0.63, −0.44)	177.45 (60.07, 310.14)	150.04 (49.71, 253.66)	−0.69 (−0.79, −0.60)
GBD Geographic Regions						
High-income Asia Pacific	12.35 (3.44, 28.08)	8.53 (2.55, 15.72)	−1.19 (−1.23, −1.15)	297.45 (78.42, 679.23)	194.47 (56.39, 356.77)	−1.36 (−1.41, −1.31)
High-income North America	12.78 (2.96, 21.53)	8.06 (1.61, 13.60)	−1.58 (−1.62, −1.54)	313.09 (67.98, 535.47)	200.96 (37.31, 345.12)	−1.49 (−1.52, −1.45)
Australasia	14.15 (3.38, 23.93)	8.56 (1.89, 14.34)	−1.77 (−1.84, −1.70)	341.18 (76.24, 579.20)	199.41 (41.75, 342.84)	−1.89 (−1.96, −1.81)
Andean Latin America	7.48 (2.63, 19.49)	6.53 (2.18, 15.09)	−0.52 (−0.62, −0.41)	182.82 (57.88, 473.70)	156.76 (47.39, 363.24)	−0.61 (−0.72, −0.50)
Tropical Latin America	8.34 (1.73, 17.34)	7.64 (1.70, 13.73)	−0.30 (−0.35, −0.26)	208.25 (39.37, 433.88)	197.23 (42.15, 356.50)	−0.25 (−0.30, −0.20)
Central Latin America	6.02 (1.59, 14.33)	5.70 (1.66, 11.50)	−0.24 (−0.36, −0.13)	145.00 (35.33, 343.55)	147.76 (40.60, 294.02)	−0.02 (−0.13, 0.09)
Southern Latin America	14.12 (3.64, 25.87)	10.84 (2.91, 18.68)	−0.66 (−0.79, −0.52)	330.80 (82.46, 611.70)	251.76 (64.38, 434.85)	−0.69 (−0.80, −0.59)
Caribbean	8.54 (3.02, 15.24)	7.94 (2.89, 13.28)	−0.10 (−0.15, −0.05)	209.38 (68.23, 382.84)	198.15 (65.72, 336.61)	−0.05 (−0.11, 0.01)
Central Europe	13.40 (3.81, 24.39)	12.13 (3.28, 20.29)	−0.45 (−0.56, −0.33)	330.41 (91.63, 603.33)	282.90 (73.39, 477.40)	−0.61 (−0.74, −0.49)
Eastern Europe	14.10 (3.79, 28.06)	10.84 (2.84, 19.01)	−1.16 (−1.28, −1.03)	379.00 (98.71, 762.46)	267.79 (67.71, 474.80)	−1.49 (−1.64, −1.34)
Western Europe	14.28 (3.31, 25.02)	9.07 (1.84, 15.50)	−1.49 (−1.52, −1.45)	331.01 (72.52, 583.72)	206.89 (39.20, 357.56)	−1.53 (−1.57, −1.49)
Central Asia	11.73 (2.44, 23.43)	5.76 (1.44, 11.34)	−2.28 (−2.38, −2.18)	322.26 (67.66, 645.06)	149.44 (36.27, 296.26)	−2.54 (−2.64, −2.44)
North Africa and Middle East	5.31 (1.79, 10.49)	4.84 (1.57, 8.62)	−0.12 (−0.26, 0.01)	136.60 (42.78, 274.45)	120.95 (36.64, 218.53)	−0.26 (−0.38, −0.14)
South Asia	4.42 (1.52, 8.02)	4.21 (1.48, 7.20)	−0.26 (−0.35, −0.17)	120.31 (39.49, 219.67)	112.31 (36.66, 193.32)	−0.33 (−0.42, −0.24)
Southeast Asia	8.41 (4.62, 13.52)	8.16 (4.23, 12.43)	−0.18 (−0.21, −0.14)	224.58 (113.04, 363.86)	211.69 (100.22, 334.64)	−0.27 (−0.30, −0.24)
East Asia	18.13 (3.86, 37.29)	8.74 (3.01, 17.81)	−2.61 (−2.73, −2.49)	462.14 (96.32, 959.07)	212.63 (71.30, 431.73)	−2.78 (−2.93, −2.63)
Oceania	6.13 (2.44, 13.16)	5.19 (1.92, 10.77)	−0.56 (−0.65, −0.47)	165.43 (57.77, 354.16)	145.69 (51.89, 302.24)	−0.42 (−0.51, −0.34)
Western Sub-Saharan Africa	3.00 (0.39, 5.58)	3.53 (0.69, 6.27)	0.79 (0.62, 0.96)	87.98 (29.19, 156.23)	103.80 (34.10, 183.58)	0.77 (0.62, 0.92)
Eastern Sub-Saharan Africa	11.33 (3.56, 18.23)	9.88 (2.93, 15.69)	−0.63 (−0.72, −0.55)	295.73 (86.46, 483.91)	239.89 (61.55, 387.71)	−0.91 (−1.00, −0.81)
Central Sub-Saharan Africa	6.77 (2.68, 11.30)	6.26 (2.81, 10.06)	−0.32 (−0.40, −0.24)	185.94 (58.73, 323.17)	167.75 (61.53, 276.15)	−0.41 (−0.49, −0.32)
Southern Sub-Saharan Africa	9.97 (2.96, 16.15)	11.74 (3.52, 19.17)	0.49 (0.15, 0.84)	260.60 (69.39, 432.43)	297.88 (83.75, 492.82)	0.44 (0.10, 0.79)

UI, uncertainty intervals; DALYs, disability-adjusted life years; ASR, age-standardized rate; EAPC, estimated annual percentage change.

### 3.3 Burden of cancers attributable to dietary risk factors at the regions defined by SDI and geographic regions

In 2021, the high-middle SDI region exhibited the highest ASDR for cancers attributable to dietary risk factors at 9.16 (95% UI: 2.87 to 16.89, per 100,000 population), along with the highest age-standardized DALYs rate of 219.57 (95% UI: 67.29 to 406.47, per 100,000). Conversely, the low-middle SDI region recorded the lowest ASDR at 5.23 (95% UI: 1.94 to 9.00, per 100,000) and the lowest age-standardized DALYs rate of 139.67 (95% UI: 46.84 to 242.91, per 100,000) (Table 1).

**Figure 3:**
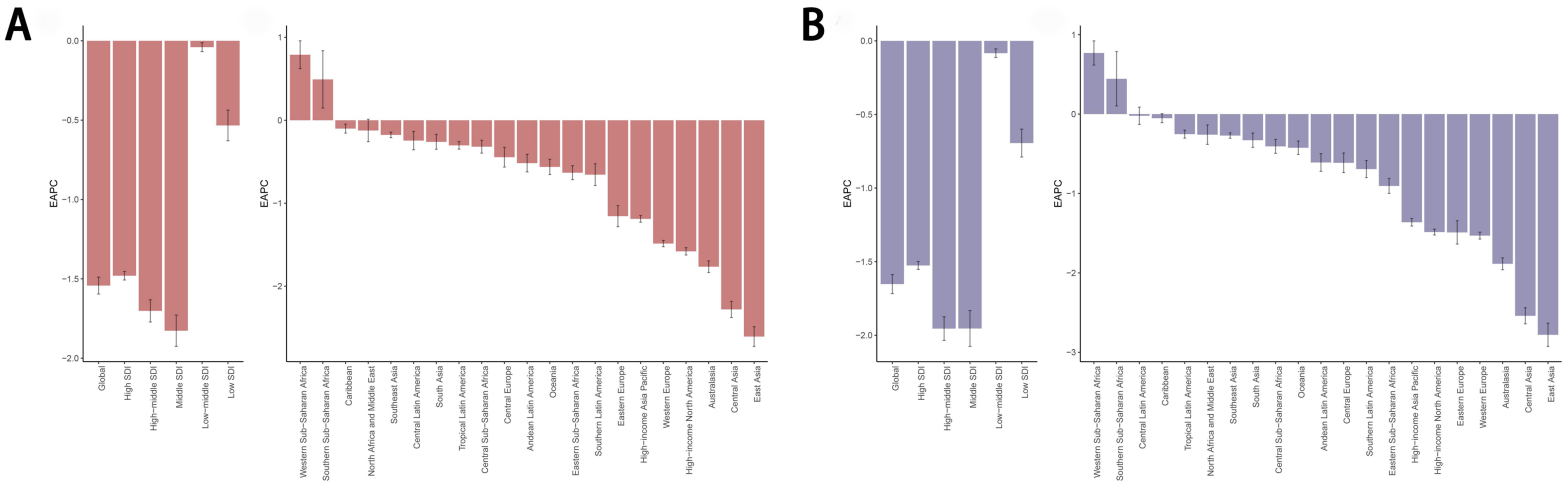
EAPC of the ASDR **(A)** and age-standardized DALYs rate **(B)** for cancers attributable to dietary risk factors in 21 Geographic Regions and 5 SDI regions from 1990 to 2021. EAPC, estimated annual percentage change; ASDR, age-standardized death rate; DALYs, disability-adjusted life years.

Among the 21 geographic regions analyzed in 2021, Central Europe had the highest ASDR for diet-related cancers at 12.13 (95% UI: 3.28 to 20.29, per 100,000), while Southern Sub-Saharan Africa showed the highest age-standardized DALYs rate at 297.88 (95% UI: 83.75 to 492.82, per 100,000). Notably, all regions except Western Sub-Saharan Africa and Southern Sub-Saharan Africa demonstrated negative trends in both the EAPC of ASDR and age-standardized DALYs rate. East Asia showed the most significant decline, with the highest EAPC values for ASDR (−2.61) and age-standardized DALYs rate (−2.78) (Table 1; Figure 3).

### 3.4 Burden of cancers attributable to dietary risk factors at the national level

**Figure 4:**
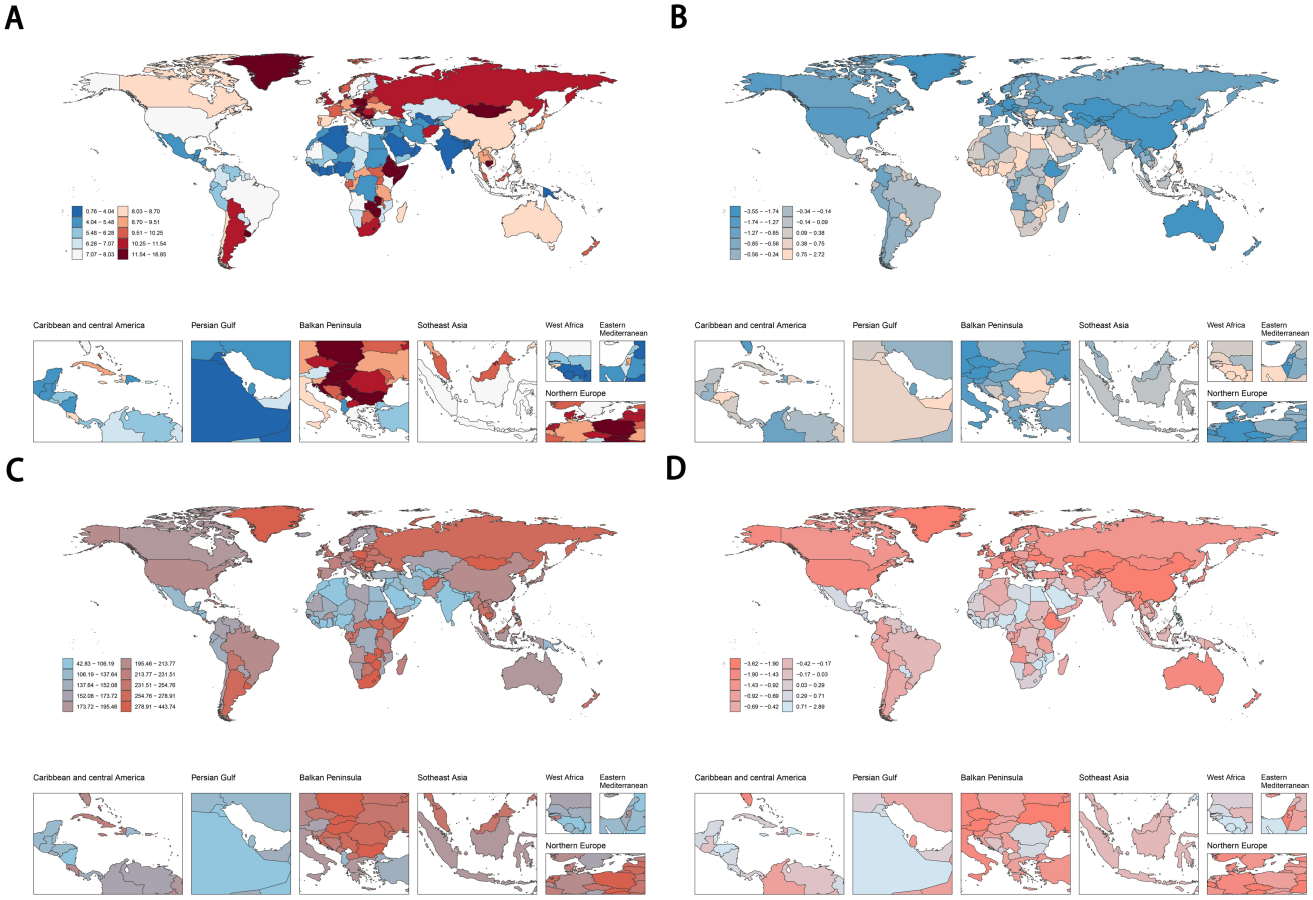
ASDR **(A,B)** and age-standardised DALYs rates **(C,D)** of cancers attributable to dietary risk factors in 2021 and their temporal trend from 1990 to 2021 at country levels. ASDR, age-standardized death rate; DALYs, disability-adjusted life years.

In 2021, Lesotho (16.69, 95% UI: 5.28 to 28.00 per 100,000), Uruguay (16.07, 95% UI: 3.66 to 27.75 per 100,000), and Zimbabwe (15.96, 95% UI: 5.93 to 25.52 per 100,000) recorded the highest ASDR for cancers attributable to dietary risk factors (Figure 4A). Similarly, Lesotho (439.34, 95% UI: 129.18 to 748.79 per 100,000), Zimbabwe (418.66, 95% UI: 143.09 to 700.07 per 100,000), and Eswatini (375.20, 95% UI: 80.70 to 693.36 per 100,000) had the highest age-standardized DALYs rates for diet-related cancers (Figure 4C). Turkmenistan demonstrated the most rapid decline in ASDR for diet-related cancers, with an EAPC of −3.52 (Figure 4B), while Kazakhstan showed the steepest decrease in age-standardized DALYs rate, with an EAPC of −3.59 (Figure 4D). Regarding specific cancer types, Malawi and Mongolia had the highest ASDR and age-standardized DALYs rate for esophageal cancer and stomach cancer attributable to dietary risk factors, respectively. Additionally, Uruguay, Indonesia, and Taiwan (Province of China) ranked highest in ASDR for colon and rectum cancer, tracheal, bronchus, and lung cancer, and breast cancer, respectively. Meanwhile, Hungary, Mongolia, and Nauru exhibited the highest age-standardized DALYs rate for colon and rectum cancer, tracheal, bronchus, and lung cancer, and breast cancer, respectively (Supplementary Figure S2).

### 3.5 The trend of cancers attributable to dietary risk factors burden from 1990 to 2021

**Figure 5:**
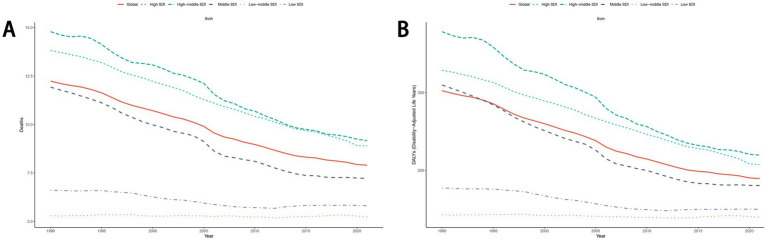
The trend of ASDR **(A)** and age-standardized DALYs rate **(B)** for cancers attributable to dietary risk factors from 1990 to 2021. ASDR, age-standardized death rate; DALYs, disability-adjusted life years.

From 1990 to 2021, the ASDR and age-standardized DALYs rate for total cancers and other diet-related malignancies exhibited a global decline. Stratified analysis across five SDI quintiles revealed that High-middle SDI regions consistently bore a disproportionately higher disease burden compared to other SDI groups (Figure 5; Supplementary Figure S3). Notably, esophageal cancer demonstrated accelerated declines in ASDR and age-standardized DALYs rate within Middle SDI and High-middle SDI regions relative to other quintiles. For diet-related stomach cancer, colon cancer, and rectum cancer, the highest ASDR and age-standardized DALYs rate were observed in High-middle SDI and High SDI regions, respectively. Conversely, breast cancer attributable to dietary risks displayed divergent trends: High-middle SDI and High SDI regions experienced downward trajectories, while Low-middle SDI, Middle SDI, and Low SDI regions showed rising burdens during this period (Supplementary Figure S3).

### 3.6 Demographic characteristics of patients with cancers attributable to dietary risk factors

**Figure 6:**
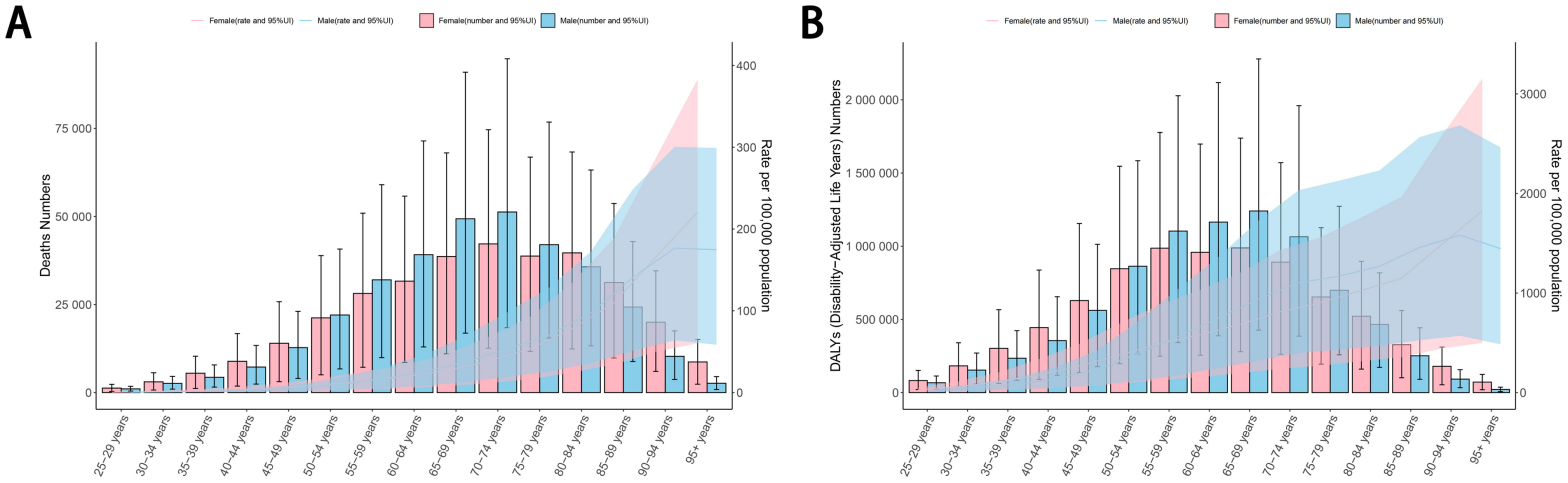
The ASDR **(A)** and age-standardized DALYs rate **(B)** per 100,000 people of cancers attributable to dietary risk factors by age and sex in 2021. ASDR, age-standardized death rate; DALYs, disability-adjusted life years.

The number of deaths and DALYs of cancers attributable to dietary risk factors was highest in the age groups of 70–74 years and 65–69 years, respectively.Among individuals aged 50–79 years, males exhibited a higher number of deaths and DALYs from diet-related cancers compared to females. For females, the ASDR and age-standardized DALYs rate for diet-related cancers increased steadily with age. In contrast, males experienced the highest ASDR and age-standardized DALY rates in the 90–94 age group (Figure 6). The disease burden of diet-related esophageal cancer, stomach cancer, and tracheal, bronchus, and lung cancer was consistently higher in males than in females. Similar to overall cancers, the ASDR and age-standardized DALYs rate for diet-related colon and rectum cancer increased with age in females, while males reached their peak rates in the 90–94 age group. Among individuals younger than 79 years, males also had a higher number of deaths and DALYs from diet-related colon and rectum cancer compared to females. For breast cancer, the number of deaths and DALYs attributable to dietary risk factors peaked in the 55–59 age group, with the ASDR and age-standardized DALYs rate increasing with age among females (Supplementary Figure S4). These findings highlight significant age- and sex-based disparities in the burden of diet-related cancers.

### 3.7 Spatiotemporal variations in cancer burden attributable to dietary factors

**Figure 7:**
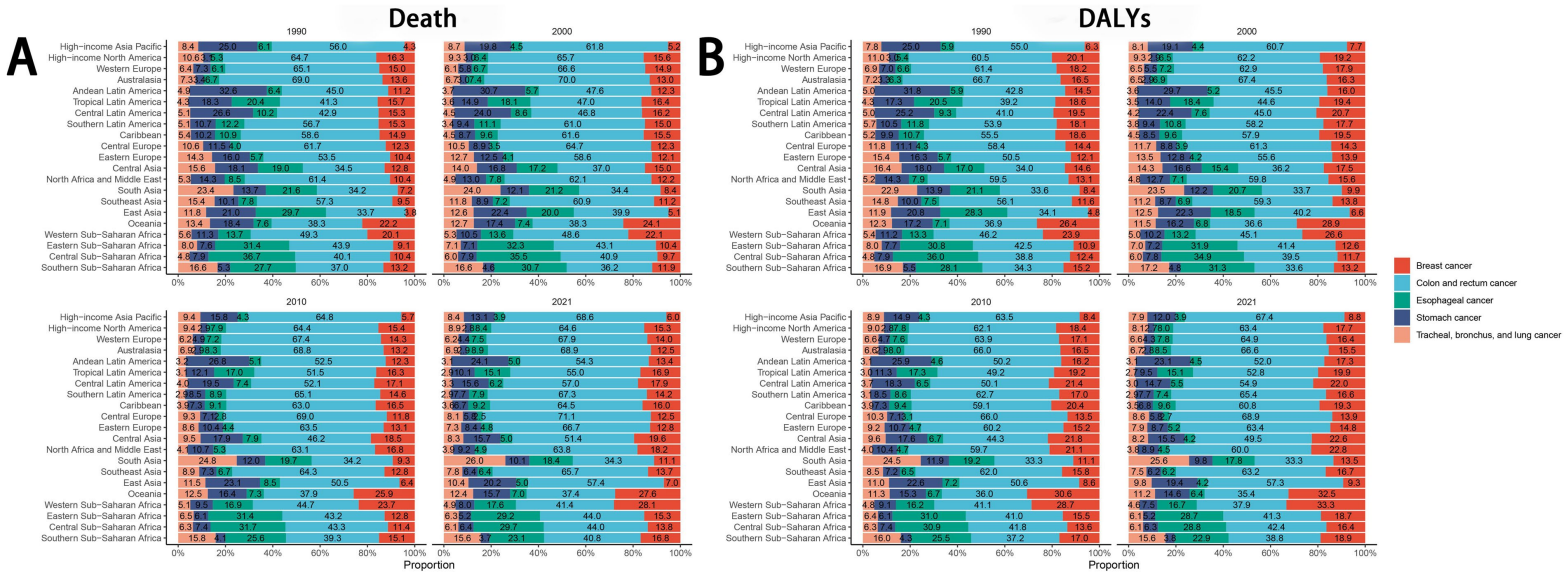
The trend of the proportion of different diseases over time.

Globally and across the 21 regions, colorectal cancer represented the largest proportion of the disease burden among all cancers attributable to dietary risk factors. According to the 2021 GBD data, regional variations were evident: esophageal cancer had the highest proportion in Central Sub-Saharan Africa, stomach cancer in Andean Latin America, colorectal cancer in Central Europe, lung cancer in South Asia, and breast cancer in Western Sub-Saharan Africa (Figure 7).

### 3.8 Dietary risk factors

**Figure 8:**
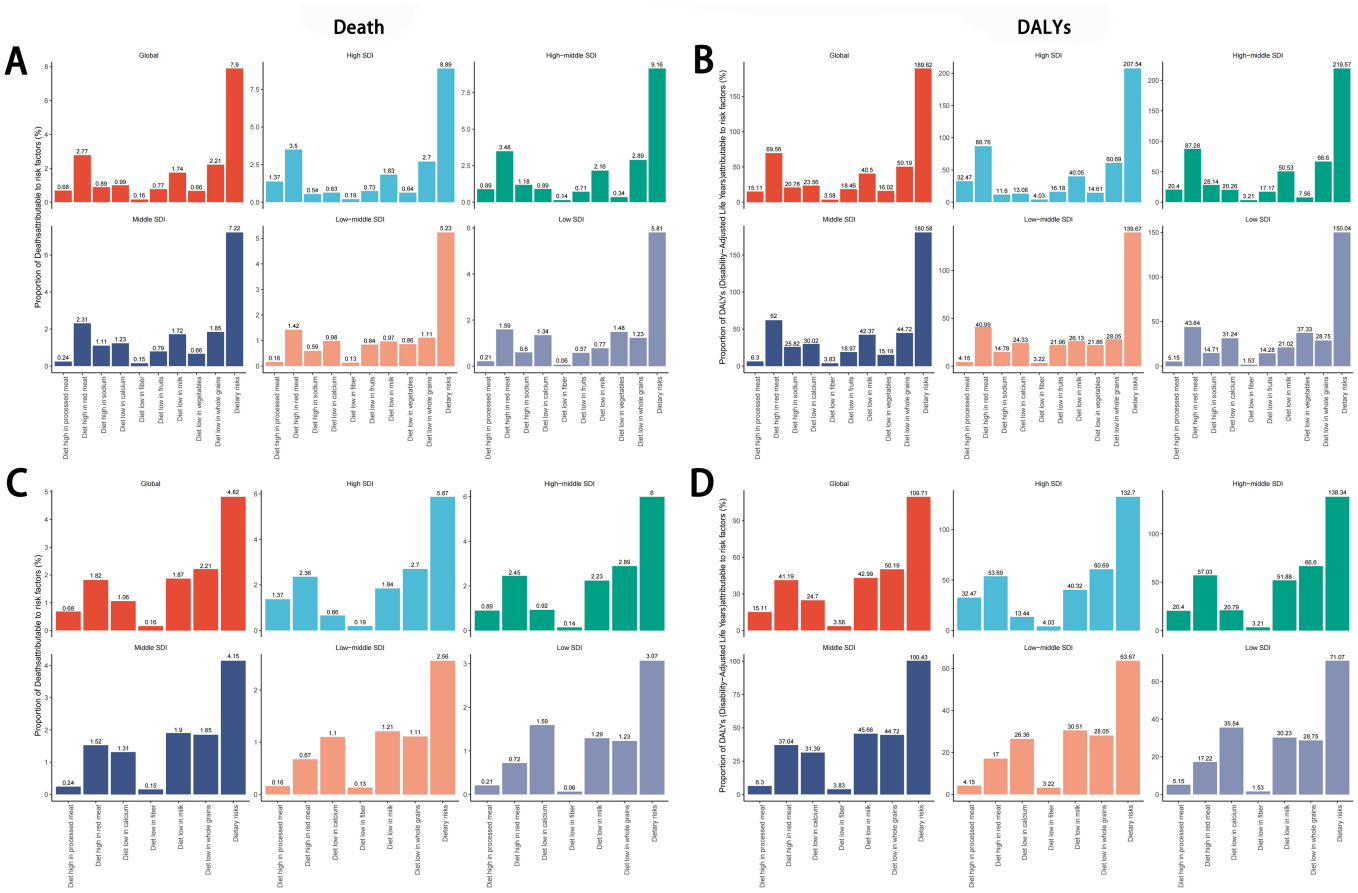
Global and regional dietary risk factors for total cancer**(A,B)** and colon and rectum cancer **(C,D)** mortality and DALYs in 2021. DALYs, disability-adjusted life years.

In 2021, high red meat intake emerged as the leading dietary risk factor for cancer-related deaths and DALYs globally and across all five SDI quintiles, while low fiber intake had the smallest impact. In all regions except low-SDI areas, insufficient whole grain consumption ranked as the second-largest contributor to cancer mortality and DALYs, followed by inadequate milk intake. In low-SDI regions, low vegetable intake was the second most influential factor, with low calcium intake ranking third (Figures 8A,B). For colorectal cancer specifically, insufficient whole grain intake was the predominant dietary risk factor in high-SDI and high-middle-SDI regions. In middle-SDI and low-middle-SDI regions, inadequate milk consumption played a larger role, while low calcium intake dominated in low-SDI regions (Figure 8C). The ranking of dietary risk factors for colorectal cancer mortality aligned closely with their contributions to DALYs (Figure 8D).

### 3.9 Relationship between burden of cancers attributable to dietary risk factors and SDI level

**Figure 9:**
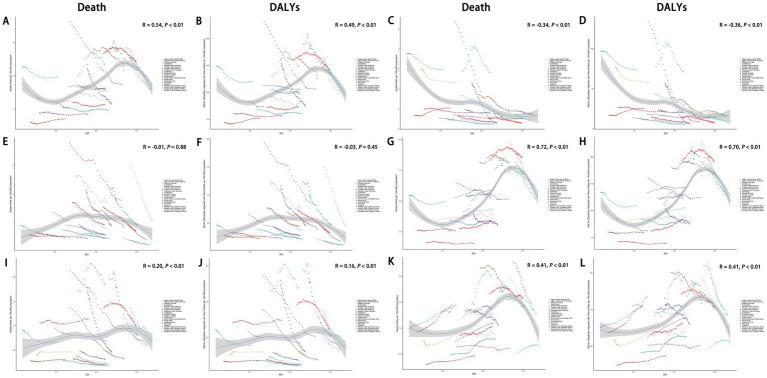
Association between ASDR and age-standardized DALYs rate of total cancers **(A,B)**, esophageal cancer **(C,D)**, stomach cancer **(E-F)**, colon and rectum cancer **(G,H)**, tracheal, bronchus, and lung cancer **(I,J)**, and breast cancer **(K,L)** attributable to dietary risk factors and SDI. ASDR, age-standardized death rate; DALYs, disability-adjusted life years; SDI, socio-demographic index.

From 1990 to 2021, across the 21 GBD regions, the ASDR for total cancers attributable to dietary risk factors showed a positive correlation with the SDI (R = 0.54, *p* < 0.01), as did the age-standardized DALYs rate (R = 0.49, *p* < 0.01) (Figures 9A,B). Similar positive correlations were observed for colon and rectum cancer, tracheal, bronchus, and lung cancer, and breast cancer attributable to dietary risk factors. In contrast, the ASDR for esophageal cancer attributable to dietary risk factors showed a negative correlation with the SDI (R = −0.34, *p* < 0.01), as did the age-standardized DALYs rate (R = −0.36, *p* < 0.01) (Figures 9C,D). In addition, the relationship between the ASDR and age-standardized DALYs rate for stomach cancer attributable to dietary risk factors and SDI was not statistically significant (Figures 9E,F).

### 3.10 Age-period-cohort analysis of diet-related cancer burden

**Figure 10:**
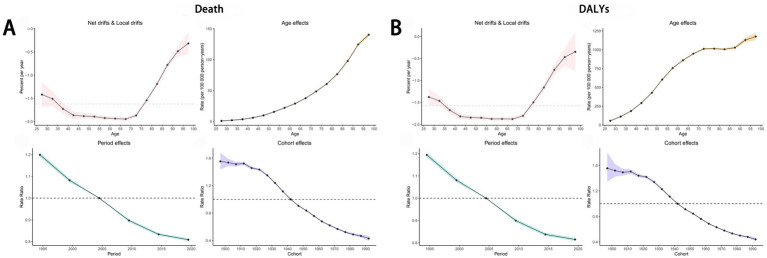
Age-Period-Cohort Analysis to estimate temporal trend of ASDR **(A)** and age-standardized DALYs rate **(B)** of cancers attributable to dietary risk factors. ASDR, age-standardized death rate; DALYs, disability-adjusted life years.

The Age-Period-Cohort (APC) analysis revealed that the net drifts for mortality and DALYs were −1.62 and −1.57, respectively, indicating a declining trend over time. For period effects, the rate ratio (RR) remained below 1.00 since 2005, suggesting a gradual reduction in the ASDR and age-standardized DALYs rate for cancers attributable to dietary risk factors. Similarly, the ASDR and age-standardized DALYs rate for diet-related cancers among individuals born after 1942 also showed a consistent decline (Figures 10A,B). The net drifts for mortality of specific cancers attributable to dietary risk factors were as follows: esophageal cancer (−3.74), stomach cancer (−2.49), colon and rectum cancer (−1.03), tracheal, bronchus, and lung cancer (−2.07), and breast cancer (−0.75). The corresponding net drifts for DALYs were −3.74, −2.49, −1.00, −2.07, and −0.69, respectively. Both period and cohort effects for these cancer types mirrored the trends observed for overall diet-related cancers (Supplementary Figure S5).

### 3.11 Decomposition analysis

To assess the contributions of population growth, aging, and epidemiological changes to trends in cancers attributable to dietary risk factors, we conducted a decomposition analysis. Results indicated that population growth and aging were key drivers of the rising burden of diet-related cancers, while epidemiological changes partially offset this increase. Notably, population growth exerted the strongest influence on the upward trajectory of age-standardized DALYs rate.

**Figure 11:**
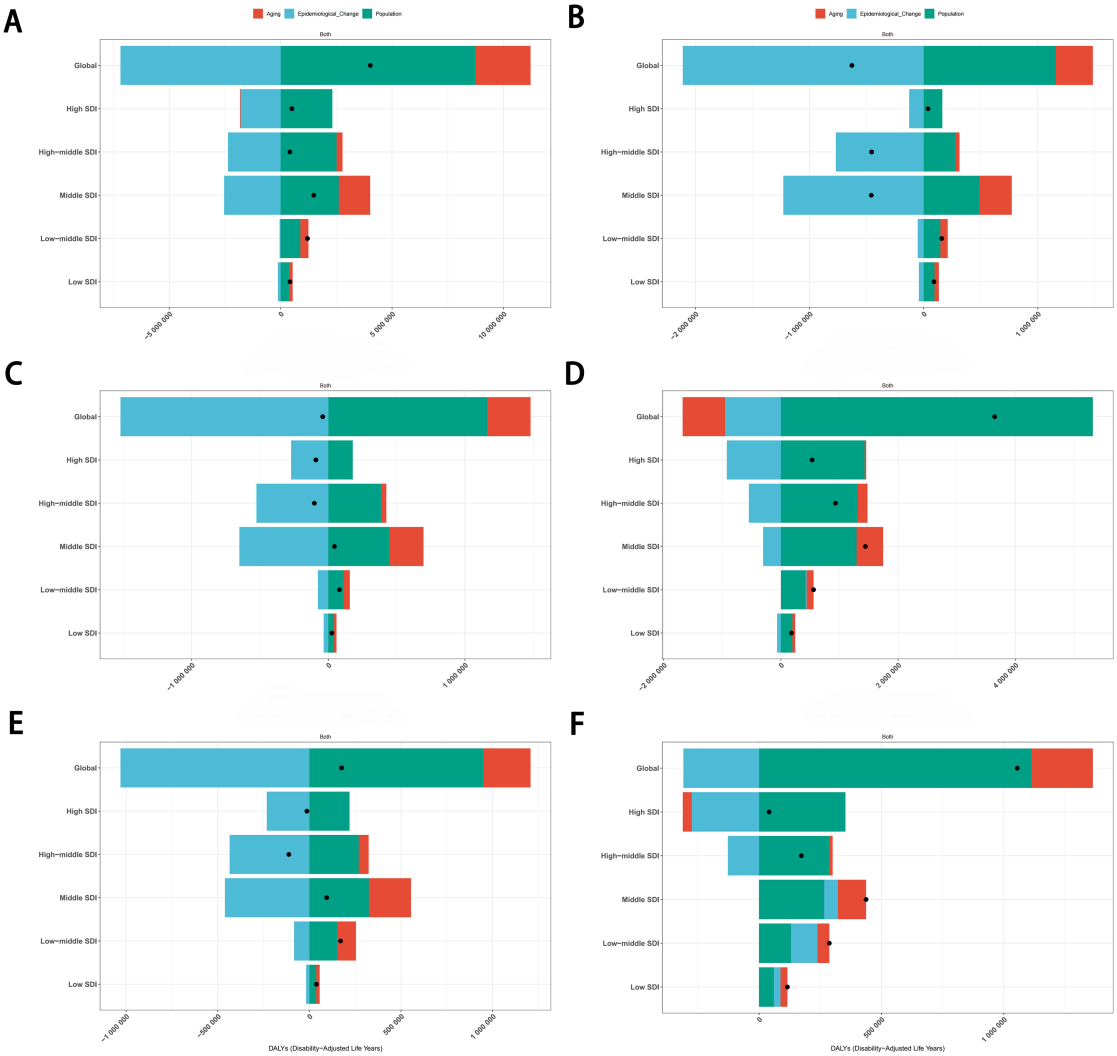
Decomposition analysis of the trends in total cancers **(A)**, esophageal cancer **(B)**, stomach cancer **(C)**, colon and rectum cancer **(D)**, tracheal, bronchus, and lung cancer **(E)**, and breast cancer **(F)** attributable to dietary risk factors age-standardized DALYs rate from 1990 to 2021. DALYs, disability-adjusted life years.

Globally, between 1990 and 2021, the overall burden of cancers attributable to dietary risk factors exhibited an upward trend across all SDI regions. Aging, population growth, and epidemiological changes contributed 61.66, −216.88%, and −178.54%, respectively, to the global increase in age-standardized DALYs rate for total cancers (Figure 11A). For colon and rectum cancer, these factors accounted for −19.84, 145.91%, and −26.07%, respectively, during the same period (Figure 11D). The divergent contributions highlight the complex interplay of demographic and epidemiological dynamics in shaping diet-related cancer burdens.

### 3.12 Health inequality analysis

**Figure 12:**
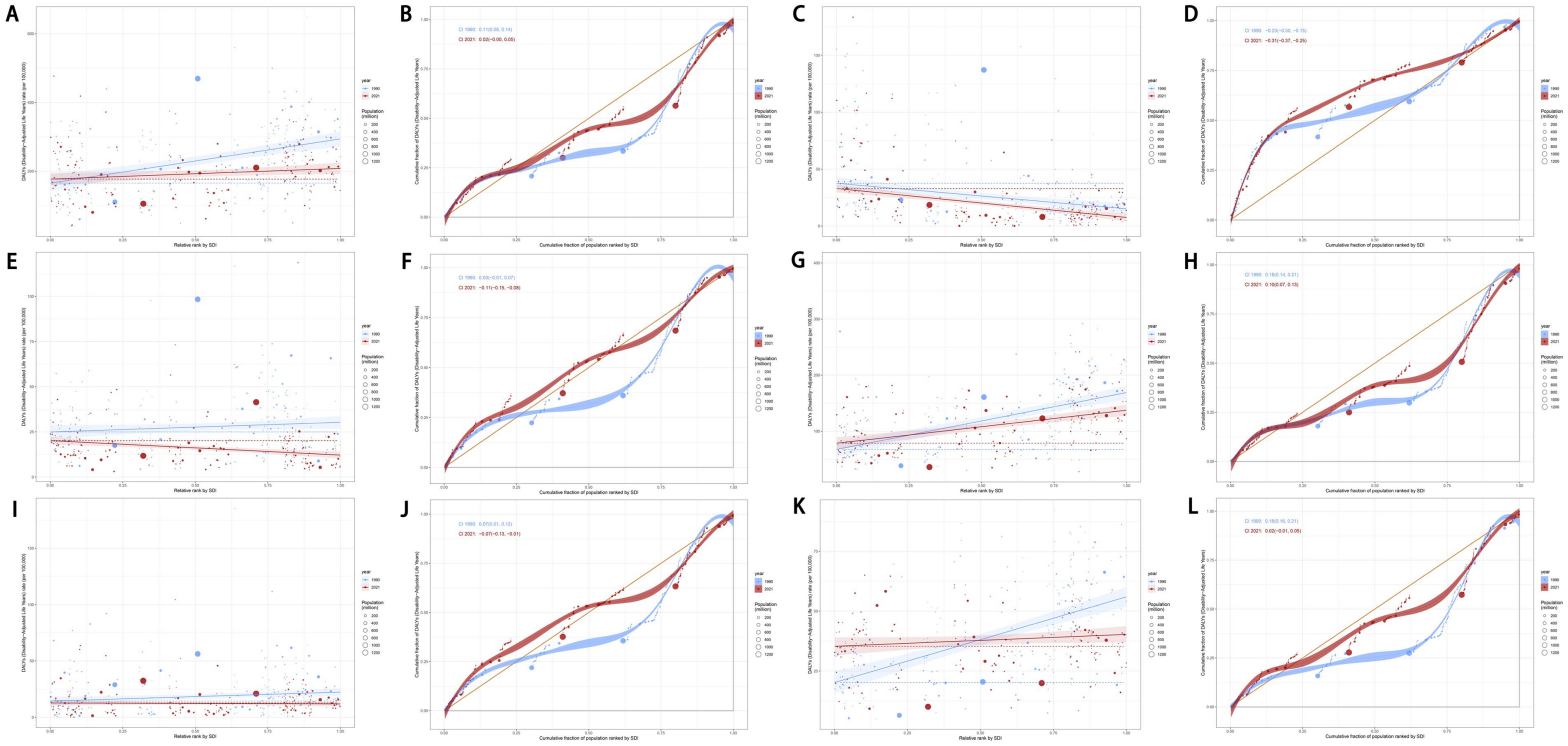
Slope indexes and concentration indexes for total cancers **(A,B)**, esophageal cancer **(C,D)**, stomach cancer **(E,F)**, colon and rectum cancer **(G,-H)**, tracheal, bronchus, and lung cancer **(I,J)**, and breast cancer **(K,L)** attributable to dietary risk factors age-standardized DALYs rate from 1990 to 2021 worldwide. DALYs, disability-adjusted life years.

Given the strong correlation between DALYs for cancers attributable to dietary risk factors and the SDI, we conducted an in-depth analysis of global health inequality. The SII for the age-standardized DALYs rate of total cancers attributable to dietary risk factors declined significantly from 128.43 (95% CI: 91.46 to 165.40) in 1990 to 31.30 (95% CI: 3.36 to 59.24) in 2021. Similarly, the CII decreased from 0.11 (95% CI: 0.08 to 0.14) in 1990 to 0.02 (95% CI: 0 to 0.05) in 2021 (Figures 12A,B). Comparable trends were observed for colon and rectum cancer, tracheal, bronchus, and lung cancer, and breast cancer.

In contrast, the SII for esophageal cancer attributable to dietary risk factors increased from −22.46 (95% CI: −30.91 to −14.01) in 1990 to −25.39 (95% CI: −31.59 to −19.19) in 2021, while the CII rose from −0.23 (95% CI: −0.30 to −0.15) to −0.31 (95% CI: −0.37 to −0.25) during the same period (Figures 12C,D). Additionally, the SII for stomach cancer attributable to dietary risk factors shifted from 5.34 (95% CI: −0.84 to 11.52) in 1990 to −7.96 (95% CI: −11.46 to −4.47) in 2021, with the CII increasing from 0.03 (95% CI: −0.01 to 0.07) to −0.11 (95% CI: −0.15 to −0.08) over the same timeframe (Figures 12E,F). These findings highlight evolving patterns of health inequality across different cancer types attributable to dietary risk factors.

### 3.13 Frontier analysis

We performed a frontier analysis to explore the optimal scenario in which countries and territories could manage their disease burden in alignment with their SDI levels. The analysis highlighted five lower-SDI countries and territories closest to the frontier fit line, marked in blue, and five higher-SDI countries and territories farthest from the frontier fit line, marked in red. Additionally, 15 countries and territories across all SDI levels that were farthest from the frontier fit line were identified and marked in black.

**Figure 13:**
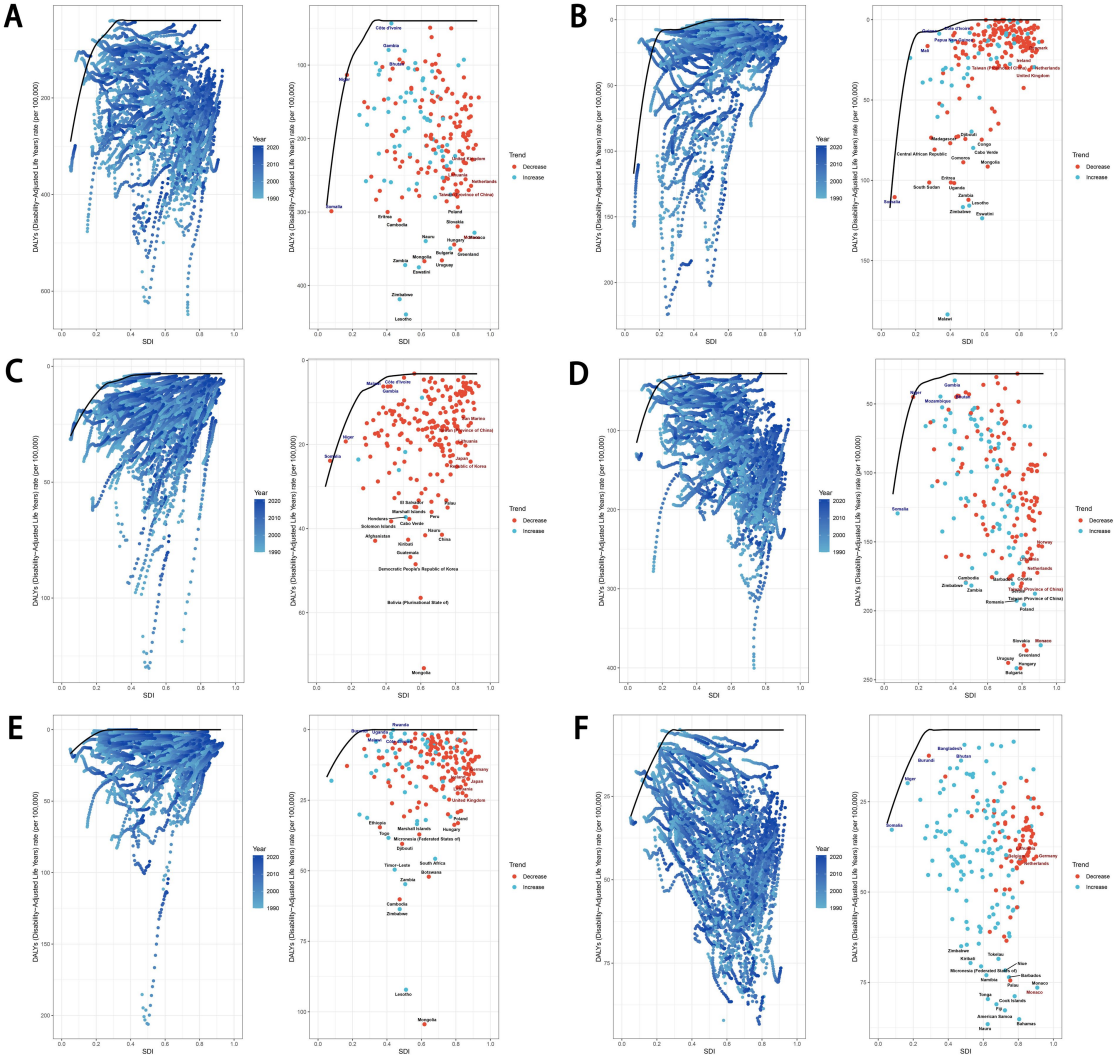
Frontier analysis exploring the relationship between SDI and age-standardized DALYs rate for total cancers **(A)**, esophageal cancer **(B)**, stomach cancer **(C)**, colon and rectum cancer **(D)**, tracheal, bronchus, and lung cancer **(E)**, and breast cancer **(F)** attributable to dietary risk factors in 204 countries and territories. SDI, socio-demographic index; DALYs, disability-adjusted life years.

For the age-standardized DALYs rate of total cancers attributable to dietary risk factors, the five lower-SDI countries and territories closest to the frontier were Somalia, Niger, Côte d’Ivoire, Gambia, and Bhutan. In contrast, the higher-SDI countries and territories farthest from the frontier line were Monaco, Taiwan (Province of China), the Netherlands, Lithuania, and the United Kingdom (Figure 13A). Similarly, for colon and rectum cancers attributable to dietary risk factors, the five lower-SDI countries and territories closest to the frontier were Somalia, Niger, Gambia, Mozambique, and Bhutan, while the higher-SDI countries and territories farthest from the frontier line were Monaco, Taiwan (Province of China), the Netherlands, Lithuania, and Norway (Figure 13D). Notably, Taiwan (Province of China) consistently ranked among the top five higher-SDI countries and territories with the farthest frontier distances in previous analyses (Figure 13). Detailed results for other cancer subtypes attributable to dietary risk factors are presented in Figure 13.

### 3.14 Projected trends in cancer mortality and DALYs attributable to dietary risks

**Figure 14:**
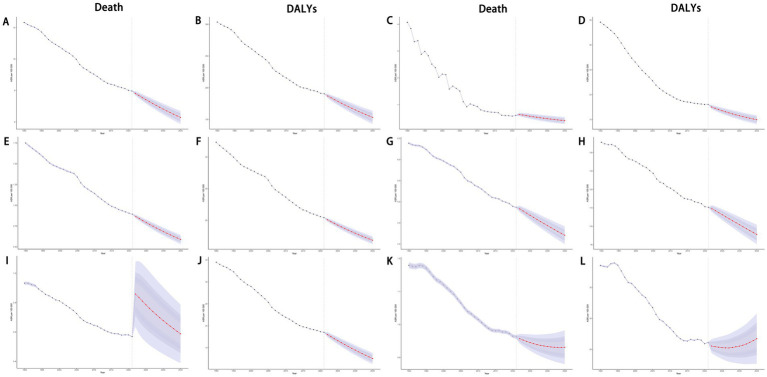
Projections of ASDR and age-standardized DALYs rate of total cancers **(A,B)**, esophageal cancer **(C,D)**, stomach cancer **(E,F)**, colon and rectum cancer **(G,H)**, tracheal, bronchus, and lung cancer **(I,J)**, and breast cancer **(K,L)** attributable to dietary risk factors by 2035. ASDR, age-standardized death rate; DALYs, disability-adjusted life years.

Using the BAPC model, we projected mortality and DALYs attributable to dietary risks for overall cancers and specific cancer types up to 2035. Globally, both mortality and DALYs rate for cancers exhibited a general decline. Similar downward trends were observed for gastrointestinal cancers, including stomach, esophageal, and colorectal cancers (Figure 14). However, breast cancer showed a contrasting pattern: while mortality rates decreased, DALYs rate increased. Further age-stratified analysis revealed that individuals under 55 years old experienced rising mortality and DALYs rate. Notably, the 55–59 age group displayed a distinct “U”-shaped trend. Additionally, lung cancer mortality surged sharply between 2020 and 2021 before gradually declining, whereas its DALYs rate consistently decreased (Figure 14; Supplementary Figure S6).

## 4 Discussion

This study combined bibliometric analysis with data from the GBD to comprehensively investigate the relationship between dietary factors and various types of cancer. Bibliometric analysis shows that over time, the number of studies on diet-related cancers has fluctuated upwards, with research cores expanding from digestive tract tumors such as gastric and colorectal cancers to other types of cancers like lung and prostate cancers. Meanwhile, there has been a growing focus on dietary fiber and red meat consumption. The emphasis on improving the quality of life for cancer patients has also significantly increased. This indicates that the study of the burden of diet-related cancers remains a current research hotspot, advancing towards more in-depth research directions, which is of great significance. Additionally, this aligns broadly with the findings from the GBD study, which identified colorectal cancer as the leading diet-related cancer and excessive red meat intake as the most significant dietary risk factor. Leveraging the most recent data from the 2021 GBD study, we conducted a comprehensive analysis of the impact of dietary risks on deaths and DALYs for various tumors at global, regional, and national levels from 1990 to 2021. Furthermore, we projected epidemiological trends up to 2035. Globally, both the ASDR and age-standardized DALYs rate for tumors attributable to dietary risks exhibited a declining trend. The disease burden was disproportionately higher among males, older populations, regions with higher SDI levels, and diet-related colon and rectum cancer. Notably, except for esophageal and stomach cancers linked to dietary risks, a significant positive correlation was observed between tumor burden and SDI levels. Using the APC model, we explored the relationship between tumor burden and age, period, and cohort effects. Frontier analyses were employed to identify countries where dietary pattern adjustments are most urgently needed. Decomposition analysis revealed that population growth was the largest positive driver of increased tumor burden, while epidemiological changes exerted a negative influence. Health inequality analysis indicated overall improvements in outcomes. According to the BAPC model, projections for 2035 suggest a declining trend in the burden of all tumors, with the exception of breast cancer DALYs rate, which are expected to rise.

Our study identified high consumption of red meat as the leading dietary risk factor for cancer. Previous research has consistently demonstrated that excessive red meat intake is associated with an increased risk of gastrointestinal tumors (20–22), lung cancer (23), and breast cancer (24). Potential mechanisms underlying this association include the high heme iron content in red meat, which promotes the formation of endogenous N-nitroso compounds in the body. These compounds can induce DNA damage through pathways such as lipid peroxidation, thereby elevating cancer risk (25). Additionally, red meat contains N-glycolylneuraminic acid, a substance that can trigger immune responses in humans and potentially promote cancer development via inflammatory pathways (26). Furthermore, a study conducted in Asian populations revealed that higher whole grain intake was associated with improved survival rates in colorectal cancer patients (HR = 0.56; 95% CI: 0.35 to 0.89) (27), suggesting that increasing grain consumption may positively influence colorectal cancer prognosis. Liliana et al. also highlighted that insufficient calcium intake might be linked to an elevated risk of breast cancer and could adversely affect patients’ nutritional status (28). Notably, our findings align with those of other studies, underscoring the importance of early dietary adjustments to mitigate the tumor burden attributable to diet (29). Among these adjustments, reducing red meat consumption emerges as a particularly urgent priority.

Except for breast cancer, the tumor burden was significantly higher in males than in females. This disparity may be attributed to males’ greater propensity for unhealthy lifestyle choices, such as smoking, heavy alcohol consumption, higher intake of red and processed meats, and relatively lower consumption of vegetables and fruits compared to females (30). Additionally, gender differences in the interaction between diet and gut microbiota, as well as variations in sex hormone levels, may also play a critical role in explaining this phenomenon (31).

In terms of the SDI, the burden of lung cancer exhibited an overall increasing trend with rising SDI values, although it declined in high SDI regions. As SDI levels increased, dietary habits shifted, with high-sugar and high-fat diets potentially elevating the risk of lung cancer (32). However, in high SDI regions, dietary patterns may have transitioned toward healthier options, contributing to the observed decline. In countries with higher economic development, changes in lifestyle and behavioral patterns, such as increased smoking rates, may have played a significant role, as smoking remains a critical risk factor for lung cancer (33). Additionally, high SDI regions likely benefited from greater health awareness and more comprehensive cancer screening programs, leading to higher detection rates of lung cancer (34). Thus, the observed trends can only be fully understood through the complex interplay of dietary, behavioral, and social factors.

The APC model analysis revealed a distinct pattern in cancer mortality and DALYs rate across different birth cohorts. Specifically, individuals born before 1942 exhibited increasing trends in both cancer mortality and DALYs rate, whereas subsequent birth cohorts demonstrated declining rates. This disparity may be attributed to the unique historical circumstances experienced by the pre-1942 cohort, who lived through the tumultuous periods of both World Wars and the Great Depression. During these times of conflict and political instability, social resources were likely diverted away from public health initiatives and personal healthcare, potentially exacerbating health outcomes. Furthermore, the economic hardships of the Great Depression led to widespread poverty, malnutrition, and limited access to medical resources, all of which are known risk factors for increased cancer incidence and mortality (35). Supporting this observation, two additional epidemiological studies have documented that the cancer burden associated with dietary factors escalates with advancing age, aligning with our research findings (36, 37). These collective insights underscore the profound impact of historical and socioeconomic factors on long-term cancer trends.

In the frontier analysis, it was observed that low SDI regions closest to the efficiency frontier could consider moderately reducing their investments in addressing dietary risks. A notable example is Somalia and Gambia, which have successfully managed to control the burden of diet-related digestive tract tumors, including esophageal, stomach, and colorectal cancers. Conversely, high SDI regions that are further from the frontier may need to increase their health economic investments to mitigate dietary risks. Indeed, all regions distant from the frontier should prioritize addressing the tumor burden associated with dietary factors. Despite their relatively high levels of economic development, regions such as Monaco, Taiwan (Province of China), the Netherlands, and Lithuania still face a significant burden of diet-attributable tumors, necessitating more urgent attention and intervention.

The decomposition analysis demonstrated that the tumor burden was predominantly driven by two key factors: population growth and epidemiological changes. While population growth contributed to an escalation in the tumor burden, epidemiological changes played a mitigating role in reducing it. Concurrently, the health inequality analysis revealed a notable improvement in health disparities related to diet-attributable tumor burdens between 1990 and 2021. Although the gap in tumor burden between low-income and high-income countries diminished, indicating a potential narrowing of wealth disparities in certain regions, persistent global inequalities in stomach and esophageal cancer burdens underscore the need for sustained, long-term attention to these critical health challenges.

The BAPC prediction model indicated a projected rise in the DALYs burden of breast cancer by 2035. This trend can be partly attributed to the escalating prevalence of obesity and overweight, which are linked to unhealthy dietary habits (38). Obesity, in particular, has been established as a significant risk factor for breast cancer (39). Additionally, dietary factors may synergize with environmental and genetic influences, collectively aggravating the breast cancer burden. Concurrently, advancements in socioeconomic conditions and heightened public health awareness have led to improved detection rates of breast cancer. This increased detection likely contributes to the observed decline in breast cancer mortality rates, even as DALY rates continue to climb.

In contrast, the mortality rate of lung cancer associated with dietary risks experienced a sharp increase from 2020 to 2021. The acceleration of population aging is a key driver of this trend. However, the COVID-19 pandemic also played a critical role by disrupting healthcare systems. The reallocation of medical resources to address the pandemic and the reduced focus on non-COVID-19 patients likely hindered the timely diagnosis and treatment of lung cancer, indirectly exacerbating lung cancer mortality during this period.

To reduce the burden of diet-related cancers, some actionable measures can be implemented. Family members can be encouraged to participate together in healthy eating plans, influencing individual behaviors by changing overall family dietary habits, such as reducing red meat consumption and increasing the proportion of vegetables, fruits, and whole grains. Community health days can also be organized to invite nutrition experts to educate the public on healthy eating. Personalized nutritional counseling services should be provided especially for the elderly. Meanwhile, government health agencies should track and record red meat consumption trends among different populations and their impact on cancer incidence, so as to adjust prevention and control strategies in a timely manner.

Our study also had the following limitations: (1) Geographic Disparities in Cancer Data Quality: Underdeveloped cancer registration systems in low-income countries may lead to underestimation or bias in mortality and other critical data. (2) Incomplete coverage of dietary risk factors: Certain potential dietary risk factors (e.g., emerging contaminants or food additives) have not been included in cancer burden research, and their long-term cumulative effects could influence future projections. (3) Uncertainty in 2035 projections: While the 2035 predictions are based on current trends, future policy interventions, technological advancements (e.g., early screening tools), or environmental changes (e.g., climate-related agricultural shifts) may significantly alter the cancer burden trajectory. However, our models did not fully account for these dynamic scenarios.

## 5 Conclusion

This study highlighted a global decline in the burden of diet-related cancers from 1990 to 2021, with projections indicating continued reductions by 2035. Colorectal cancer accounted for the largest disease burden, followed by breast cancer. For most cancers, excluding esophageal and stomach cancers, the burden was positively correlated with SDI levels. However, males, older populations, and regions with higher SDI continued to bear a disproportionate share of the burden. Targeted dietary policies and SDI-stratified interventions were needed to address high-risk groups.

## Data Availability

The original contributions presented in the study are included in the article/Supplementary material, further inquiries can be directed to the corresponding author.

## Footnotes

^1^ http://ghdx.healthdata.org/gbd-results-tool/
