# Supplementary material for: Global impact of dietary risks on cancers: burdens across regions from 1990 to 2021 and the projection to 2035
Source: Front Nutr. 2025 Jul 4;12:1585305. doi: 10.3389/fnut.2025.1585305 (PMC12271164; doi:10.3389/fnut.2025.1585305)
Supplement: Supplementary file 2 [file Image_1.pdf]

Supplementary figures:

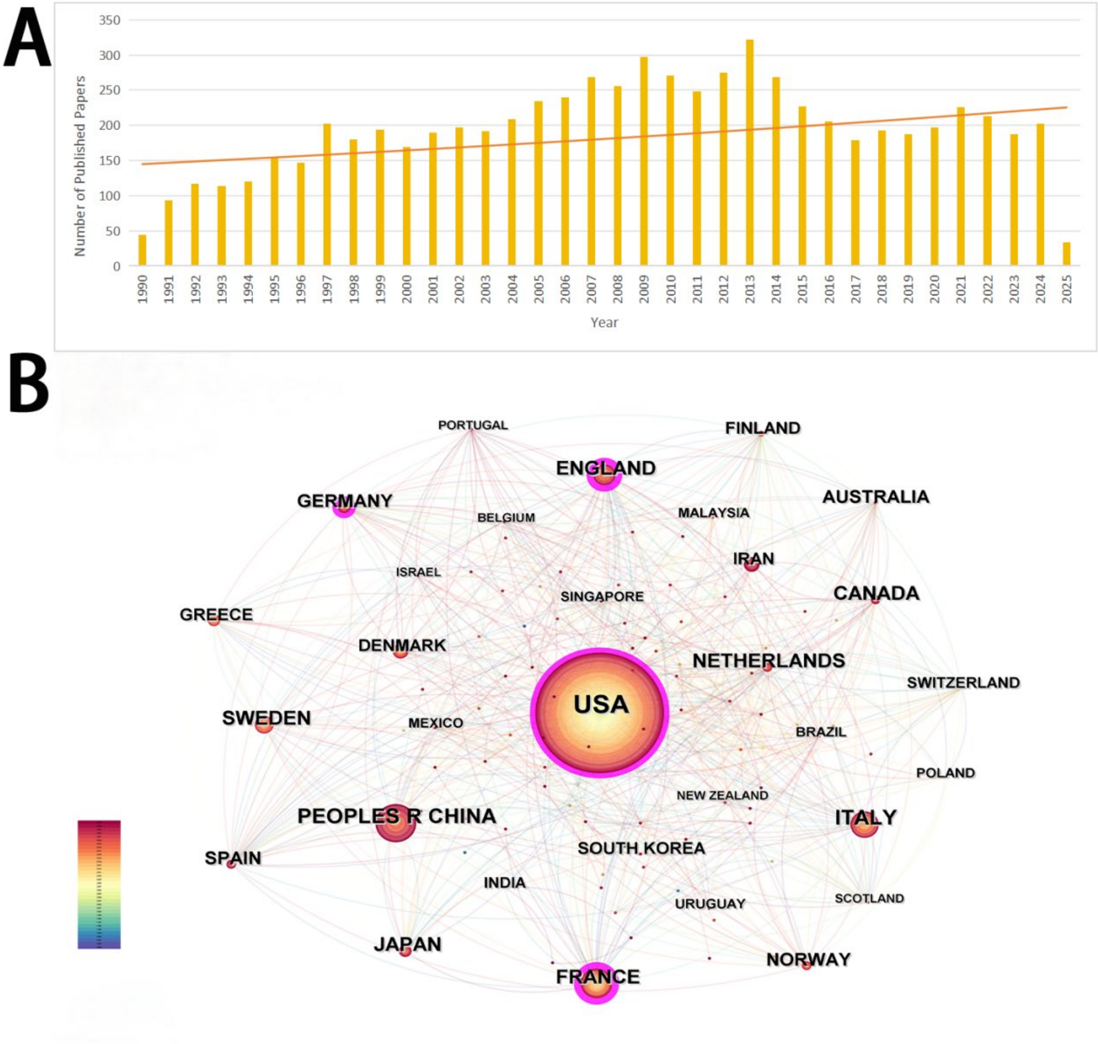

Fig. S1: Bibliometric analyses results. (A) Annual publication volume and trends. (B) Networks of country cooperation.

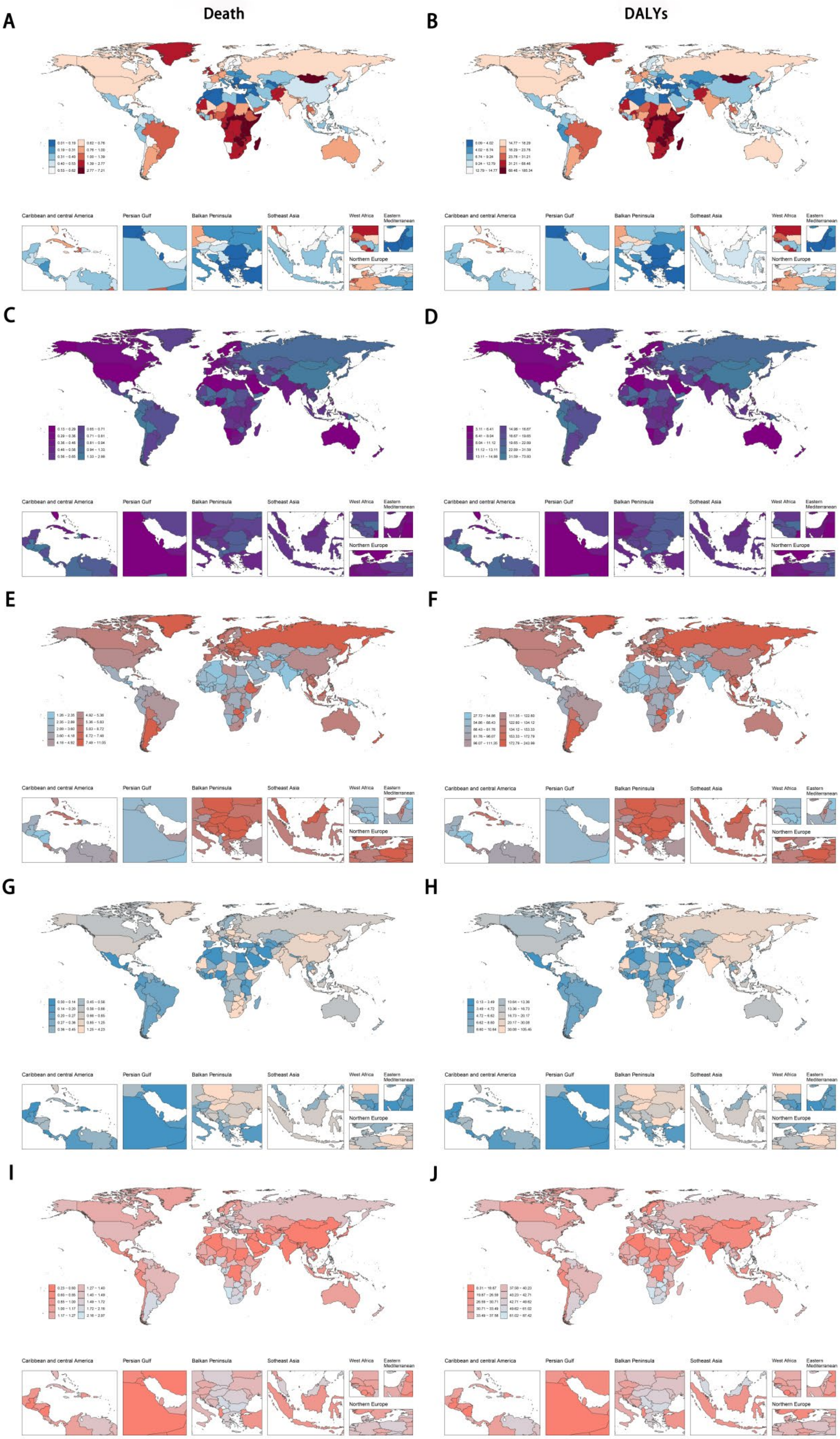

A
Death
B
DALYs
C
D
E
F
G
H
I
J
Caribbean and central America
Persian Gulf
Balkan Peninsula
Sotheast Asia
West Africa
Eastern Mediterranean
Northern Europe

Fig. S2: ASDR and age-standardised DALYs rates of esophageal cancer (A-B), stomach cancer (C-D), colon and rectum cancer (E-F), tracheal, bronchus, and lung cancer (G-H), and breast cancer (I-J) attributable to dietary risk factors in 2021 at country levels. ASDR, age-standardized death rate; DALYs, disability-adjusted life years.

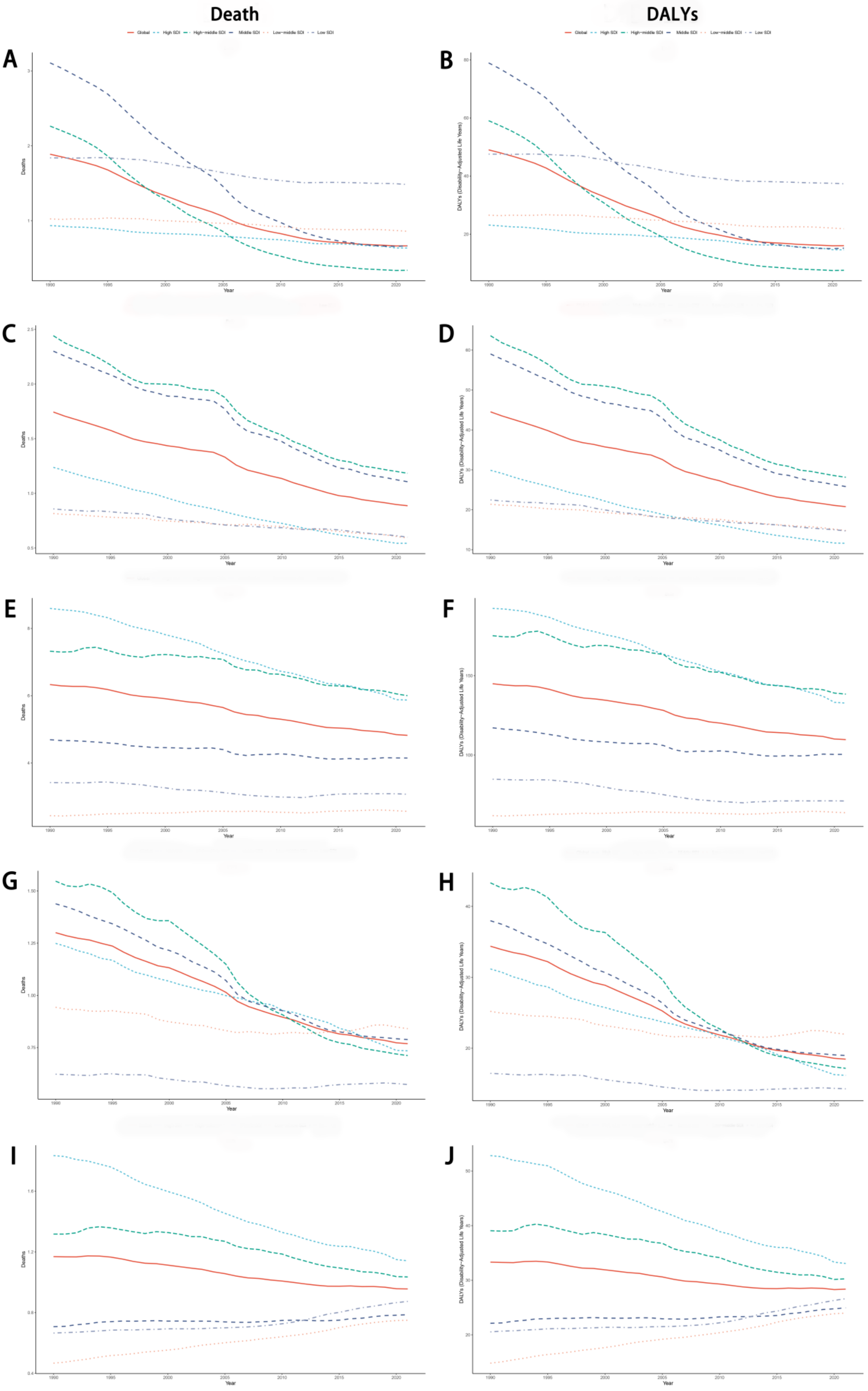

Fig. S3:The trend of ASDR and age-standardized DALYs rate for esophageal cancer (A-B),

stomach cancer (C-D), colon and rectum cancer (E-F), tracheal, bronchus, and lung cancer (G-H), and breast cancer (I-J) attributable to dietary risk factors from 1990 to 2021. ASDR, age-standardized death rate; DALYs, disability-adjusted life years.

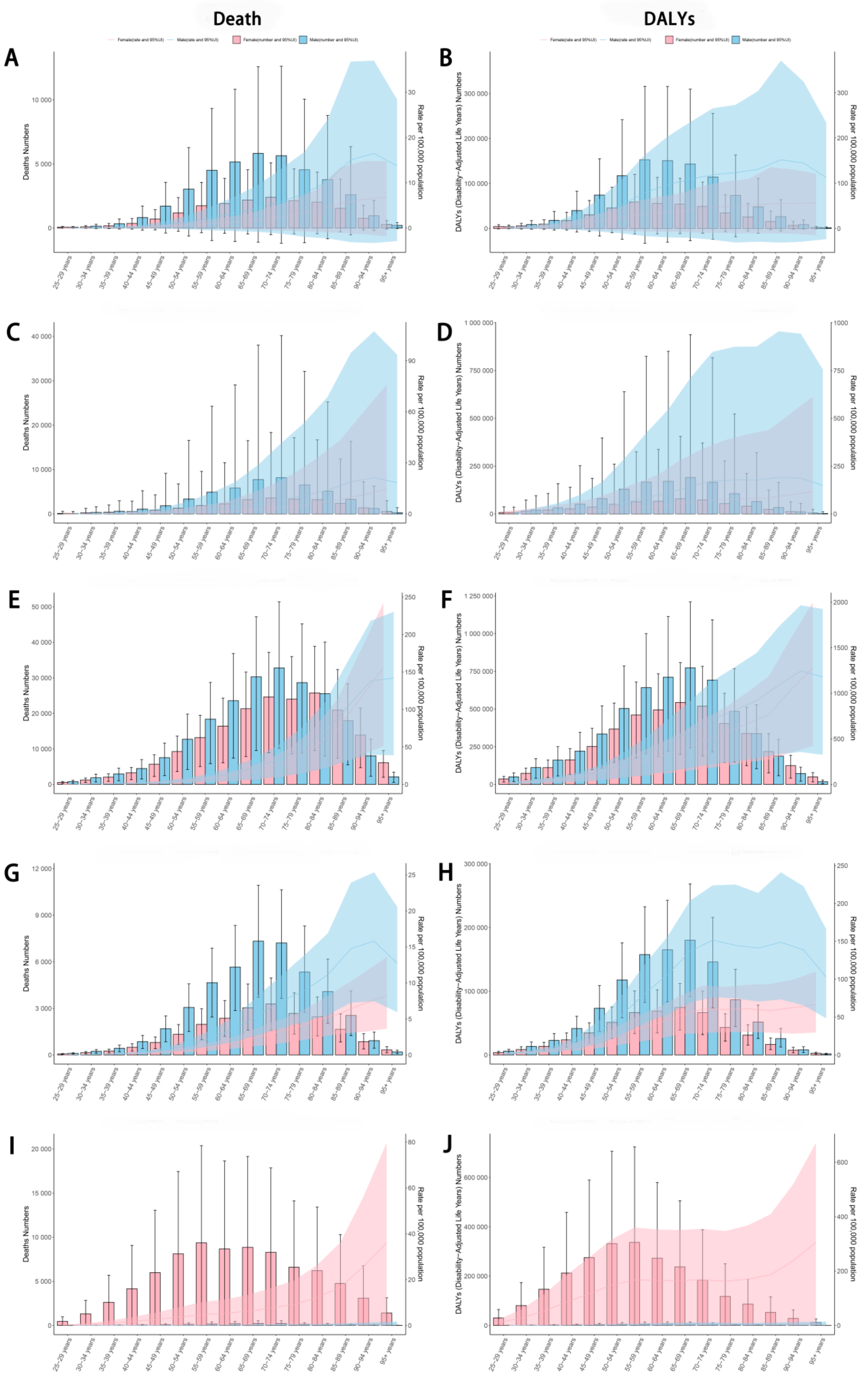

Fig. S4:The ASDR and age-standardized DALYs rate per 100,000 people of esophageal cancer

(A-B), stomach cancer (C-D), colon and rectum cancer (E-F), tracheal, bronchus, and lung cancer (G-H), and breast cancer (I-J) attributable to dietary risk factors by age and sex in 2021. ASDR, age-standardized death rate; DALYs, disability-adjusted life years.

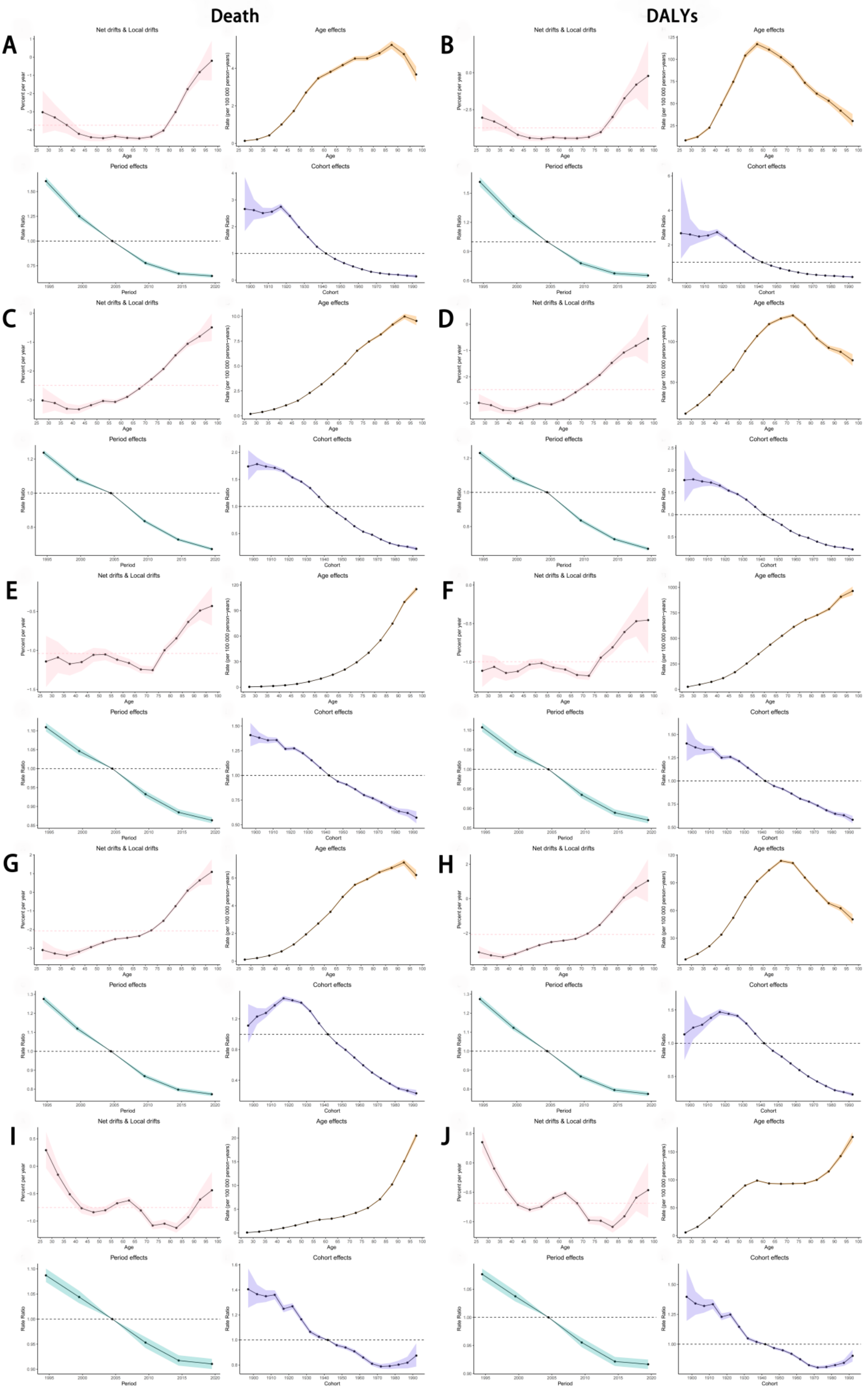

Fig. S5: Age-Period-Cohort Analysis to estimate temporal trend of ASDR and age-standardized

DALYs rate of esophageal cancer (A-B), stomach cancer (C-D), colon and rectum cancer (E-F), tracheal, bronchus, and lung cancer (G-H), and breast cancer (I-J) attributable to dietary risk factors. ASDR, age-standardized death rate; DALYs, disability-adjusted life years.

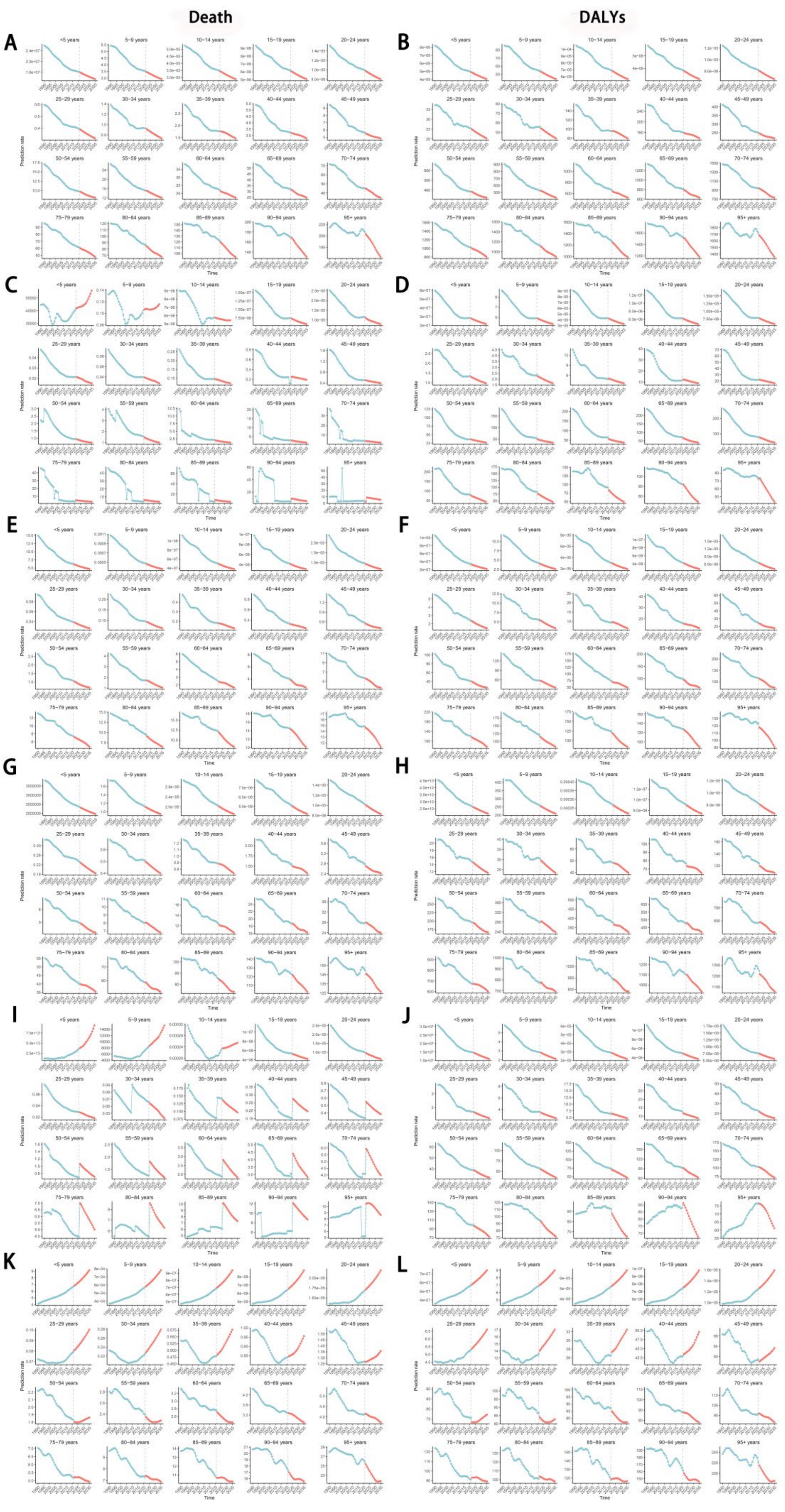

Death
DALYs
A
B
C
D
E
F
G
H
I
J
K
L
<5 years
5–9 years
10–14 years
15–19 years
20–24 years
25–29 years
30–34 years
35–39 years
40–44 years
45–49 years
50–54 years
55–59 years
60–64 years
65–69 years
70–74 years
75–79 years
80–84 years
85–89 years
90–94 years
95+ years
Prediction rate
Time

Fig. S6: Projections of ASDR and age-standardized DALYs rate of total cancers (A-B), esophageal cancer (C-D), stomach cancer (E-F), colon and rectum cancer (G-H), tracheal, bronchus, and lung cancer (I-J), and breast cancer (K-L) attributable to dietary risk factors by 2035, based on age characteristics. ASDR, age-standardized death rate; DALYs, disability-adjusted life years.
